# A reversible cell penetrating peptide-cargo linkage allows dissection of cell penetrating peptide- and cargo-dependent effects on internalization and identifies new functionalities of putative endolytic peptides

**DOI:** 10.3389/fphar.2022.1070464

**Published:** 2022-11-21

**Authors:** Daniel P. Morris, Lucy C. Snipes, Stephanie A. Hill, Michael M. Woods, Maria M. Mbugua, Lydia R. Wade, Jonathan L. McMurry

**Affiliations:** Department of Molecular and Cellular Biology, Kennesaw State University, Kennesaw, GA, United States

**Keywords:** cell-penetrating peptides, protein transduction domains (PTD), endolytic peptides, endocytosis, calmodulin (CAM)

## Abstract

Cell penetrating peptides (CPPs) are a promising technology for therapeutic delivery of macromolecular cargos. CPPs have generally used covalent linkages to cargo, ensuring a common fate as one molecule. Conversely, our CPP-adaptor, TAT-CaM, noncovalently binds calmodulin binding sequence (CBS)-containing cargos in calcium rich media then dissociates in the calcium-poor endosomal environment following internalization, enhancing endosomal escape relative to standard CPPs. In this study, we report cell entry of positively charged protein cargos that were not increased by TAT-CaM while cargos based on the negatively charged maltose binding protein (MBP) displayed little intrinsic internalization but were internalized by TAT-CaM. In addition, association of positively charged proteins with negatively charged nucleic acids reduced internalization. This evidence points to the dominant role cargo charge plays in apparent CPP effectiveness. There has been little systematic investigation as to how interaction between CPPs and cargos impacts internalization efficiency. Our adaptors provide a tool that allows combinatorial assays to detect emergent properties. Toward this end we added 4 endolytic peptide (EP) sequences between cargo CBS and MBP moieties to create 4 new cargos and between TAT and CaM to create 4 new adaptors. The new cargos were assayed for internalization alone and with a panel of CPP-adaptors to identify combinations that displayed increased internalization efficiency or other properties. Among the most important results, addition of the EP LAH4 improved adaptor performance and provided some CPP capability to cargos. MBP-LAH4-CBS was internalized more effectively by most adaptors, suggesting this sequence has general stimulatory ability. Two other EPs, Aurein 1.2 and HA2, also provided some CPP capability to their MBP cargos but were unexpectedly antagonistic to internalization by most adaptors due to retention of adaptor/cargo complexes on the cell surface. We thus identified LAH4 as stimulator of internalization in both adaptors and cargos and uncovered new functionality for Aurein 1.2 and HA2, which may be related to their identification as EPs. Future experiments will test new endolytic capabilities made possible with combinatorial approaches.

## Introduction

Cell-penetrating peptides (CPPs), sometimes called protein transduction domains (PTDs), have long held great promise for overcoming failures of biomolecule therapeutic leads due to bioavailability issues relating to failure to cross membranes (Fonseca et al., 2009; Johnson et al., 2011; Lönn and Dowdy, 2015; Derakhshankhah and Jafari, 2018). Though the specific endocytic mechanisms by which they do so remain a subject of debate, CPPs are capable of mediating penetration of the plasma membranes of mammalian cells by molecules to which they are coupled, allowing delivery of “cargos” to cell interiors, a potentially transformative platform technology for drug delivery and other applications. However, development of CPP-based delivery systems has been hindered because cargos are normally coupled to CPPs by covalent or other irreversible linkages and become entrapped in endosomes after cellular entry rather than reaching the cytoplasm and their subsequent targets (Lecher et al., 2017). To address this problem, we have employed a high affinity, reversible noncovalent coupling strategy, attaching cargos to CPPs *via* a CPP-containing “adaptor” protein, calmodulin. Our prototype CPP-adaptor, TAT-Calmodulin (TAT-CaM), consists of the cell penetrating moiety from HIV transactivator of transcription and human calmodulin (Salerno et al., 2016; Ngwa et al., 2017). TAT-CaM binds CaM binding-site (CBS) containing cargos with nM affinity in the presence of calcium but negligibly in its absence. The CBS can be located anywhere in the cargo. TAT-CaM/cargo complexes form spontaneously in the Ca^2+^-containing extracellular milieu but, concurrent with Ca^2+^ flux during early endocytosis (Albrecht et al., 2015), cargos dissociate from CPP-adaptors within endosomes and are released to the cytoplasm though the CPP-adaptor remains trapped.

Our first reports of the success of our reversible coupling strategy (Salerno et al., 2016; Ngwa et al., 2017) were attended by high hopes for a generally utile delivery vehicle adaptable not only to any desired protein but nucleic acids and perhaps other biomolecules as well. Possible cargos are limited only by the requirement of attaching a CBS to the cargo, which can be achieved recombinantly for proteins, with covalent chemistry for nucleic acids, or *via* a secondary adaptor strategy such as using a recombinant CBS-biotin binding protein to deliver biotinylated nucleic acids. In some respects, those hopes were realized as we showed that TAT-CaM worked for multiple model cargo proteins in all cell lines that we examined. Subsequent efforts to develop CPP-based applications ensued, some of which were very successful, e.g. we delivered human papillomavirus E2 protein to cervical cancer cells, inducing senescence and thus demonstrating that cargos remained folded and active even after delivery (LeCher et al., 2020).

In the course of efforts to develop applications, particularly co-delivery of Cas9 and crRNAs to effect CRISPR/Cas genome editing by exogenous delivery of the components, we noticed a correlation between charge of the cargo and efficacy of TAT-CaM-mediated delivery, the more negative the cargo, the worse the delivery. We also observed that positively charged cargo proteins were taken up themselves, sometimes as well as or even better than when coupled to TAT-CaM. Noting that most CPPs are positively charged and calmodulin is itself rather extensively negatively charged (net −24, TAT-CaM with its positive CPP and linker sequences has a charge of −16), we sought to develop next-generation CPP-adaptors that might better utilize more favorable charge states to deliver cargos, the first of which incorporated calmodulin from naked mole rat, *Heterocephalus glaber*. Naked mole rat CaM (NMR-CaM) has a positively charged domain of unknown function N-terminal to the EF hand domain, which is invariant from that of human CaM. The resultant adaptor, TAT-NMR-CaM, has a lesser net negative charge (−5) and evinced exactly the behavior we predicted, delivering cargos that had resisted efficient TAT-CaM-mediated delivery, e.g. polyA binding protein PAB-1 (Gentry et al., 2021).

Although not a basis for early adaptor design, we realized that the CPP-adaptor strategy afforded the ability to directly compare internalization of cargo alone with that of the CPP-adaptor/cargo complex, an advantage over examinations of cargo charge done with traditional covalent coupling strategies, e.g (Hymel et al., 2022). It was the availability of this comparison that led to the realization that cargo charge dramatically changed the properties of TAT-CaM/cargo complexes. In support of this idea we report herein a body of experimental data using positive and negative cargos as well as adaptors designed to have a more positive net charge.

Another advantage of the adaptor/cargo model is the ability to change the characteristics of the adaptor and the cargo independently. This allows for the improved CPP characteristics of new adaptors without impact on the released cargos’ ability to leave the endosome. Similarly, adaptor delivery systems can be modified to effect more efficient cytoplasmic delivery of cargos by inclusion of endolytic peptide sequences (EPs) (also called “endosomolytic peptides” or “endosomal escape domains”)(Aqeel and Khan, 2022). Some EPs are thought to destabilize endosomes in a pH-dependent manner by binding and disrupting membranes during the acidification that occurs in early transport, e.g. haemagglutinin-derived peptides (Wharton et al., 1988). Toward this end we added four endolytic peptide (EP) sequences: Aurein 1.2, GALA, HA2 and LAH4 (Wadia et al., 2004; Akita et al., 2011; Shahmiri et al., 2015; Moulay et al., 2017) to cargos between the CBS and negatively charged maltose binding protein (MBP) to create 4 new cargos and between TAT and CaM to create 4 new adaptors to assess internalization as a function of adaptor and cargo charge. Because both CaM and MBP have intrinsic negative charge, neither have significant intrinsic internalization behavior, and the effects of the EPs are manifest without complication from competing behaviors. While EPs are supposed to increase escape, an increase in intracellular concentration may also be due to advantageous CPP-like behavior. In this report we have focused on the clear and differential impact these EPs have on internalization.

## Materials and methods

### Plasmids, strains, and cell lines

The *E. coli* strain used in this study, BL21 (DE3)pLysS was propagated from purchased cells from EMD Millipore (Burlington, MA, United States) or other established supplier. Baby hamster kidney (BHK) cells (#CCL-10) and HEK-293 cells (CRL-1573) were purchased from ATCC and cultured in Dulbecco’s Modified Eagles’ Medium with GlutaMAX Supplement (Gibco, United States) and 5% or 10% fetal bovine serum.

Plasmids used in this study were previously described (Salerno et al., 2016; Ngwa et al., 2017; Gentry et al., 2021) or constructed as described in Supplementary Figure S1 from parent vectors pET19b and pET22b (EMD Millipore, United States), pMalc-5x (New England Biolabs, United States) or pCal-N-FLAG (Agilent Technologies, United States). For example, pJM161, encoding TAT-naked mole rat calmodulin (TAT-NMR-CaM) consisted of an *E. coli*-optimized synthetic gene (GeneScript, Piscataway, NJ, United States) cloned into NdeI and BamHI sites in pET19b with an in-frame stop codon prior to the BamHI site. The encoded TAT-NMR-CaM protein consists of the TAT peptide sequence (YGRKKRRQRRR) N-terminally fused to *Heterocephalus glaber* (naked mole rat) calmodulin (GenBank: EHB02604.1) (Gentry et al., 2021). A vector-encoded 10xHis tag containing an enterokinase cleavage site and several spacer residues is N-terminal to TAT. The version of TAT-CaM used in this study, initially designated “TAT-CaM 2.0” (Ngwa et al., 2017), is a variant of a prior version modified to eliminate some extraneous sequences originally designed to facilitate cloning; however, the His-tag, TAT and CaM sequences are invariate from the original study (Salerno et al., 2016). All references to TAT-CaM in this paper mean TAT-CaM 2.0.

### Expression, purification, and labeling

Proteins were expressed essentially as described (Salerno et al., 2016; Ngwa et al., 2017) with minor modifications. Briefly, plasmids were freshly transformed into BL21 (DE3)pLysS. Overnight cultures grown from single colonies were subcultured into 1L Luria-Bertani broth and grown with vigorous shaking at 37°C. At OD_600_ ∼0.4, the temperature was lowered to 30°C, cells were induced with 0.2 mM IPTG and growth continued for 4 hours. The procedure was altered slightly for MBP-CBS cargos, the cells for which were grown in Terrific Broth (TB) supplemented with 0.25% (w/v) glucose and induction was conducted at 32°C. Cells were harvested by centrifugation at 10,000 × g and frozen at −80°C.

Purification was also performed essentially as described *via* immobilized metal affinity chromatography (Salerno et al., 2016). Briefly, cell pellets were thawed on ice, resuspended in lysis buffer (50 mM Tris pH 8, 500 mM NaCl, 10 mM imidazole, 10% glycerol and 6 mM β-mercaptoethanol for disulfide containing proteins). 1 mg/ml DNAse and 0.25 mg/ml lysozyme were added during resuspension. For TAT-NMR-CaM only, Halt Protease Inhibitor Cocktail (ThermoFisher, Waltham, MA, United States) was added to 1x per manufacturer’s protocol. Cells were broken *via* passage through a French press at 20,000 psi and subjected to centrifugation at ∼27,000 × g to pellet unbroken cells and debris. Clarified lysate was passed over a cobalt affinity column using an FPLC system while monitoring A_280_, washed with wash buffer (equivalent to lysis buffer with 25 mM imidazole instead of 10 mM) until baseline absorbance was attained, after which protein was eluted in elution buffer (lysis buffer with 250 mM imidazole). Protein-containing fractions were pooled, concentrated and exchanged by passage over a desalting column into 10 mM HEPES, pH 7.4, 150 mM NaCl, 10% glycerol, 1 mM CaCl_2_ for fluorescence labeling, biotinylation or other further use. Quantitation was done using Bradford Assay with bovine serum albumin as standard. For fluorescence labeling, DyLight 488, 550 or 650 NHS Esters (ThermoFisher) were used to introduce fluorescent labels under conditions that resulted in incorporation efficiencies below 0.6 dyes/molecule as determined using the algorithm suggested by the manufacturer (ThermoFisher). Dye removal columns were then used to remove unreacted dye as recommended by the manufacturer (ThermoFisher).

All proteins were assayed by biolayer interferometry to assure high affinity (low nM-to-high pM) binding between adaptors and CBS-cargos in the presence of calcium and fast dissociation upon its removal with EDTA. All evinced kinetics and affinities highly similar to CaM with a natural binding partner (McMurry et al., 2011) and within the ranges observed for previously reported adaptor-cargo pairings (Salerno et al., 2016; Ngwa et al., 2017; Gentry et al., 2021). Table 1 lists all adaptors and cargos used in this study and lists the plasmids from which they were expressed.

**TABLE 1:** Schematic descriptions of proteins used in this study; A, CPP-adaptors and adaptor controls; B, cargo proteins. Additional details including amino acid sequences, spacers and tags are described in Supplementary Information.

A	Name	Description (N-to-C)	Parent vector	References
	TAT-CaM (2.0)	His-TAT-CaM	pET19b	Ngwa et al. (2017)
	CaM Cntl	His-CaM (no TAT)	pET19b	This study
	TAT-Aur-CaM	His-TAT-Aurein 1.2-CaM	pET19b	This study
	TAT-GALA-CaM	His-TAT-GALA-CaM	pET19b	This study
	TAT-LAH4-CaM	His-TAT-LAH4-CaM	pET19b	This study
	TAT-HA2-CaM	His-TAT-HA2-CaM	pET19b	This study
	TAT-NMR-CaM	His-TAT-naked mole rat CaM	pET19b	Gentry et al. (2021)
	pGFP-CaM	His-GFP (+36)-CaM	pET19b	Lawrence et al. (2007), This study
B				
	CBS-Tam	CBS-Tamavidin (C627S)-His	pET22b	This study
	MBP-CBS	MBP-CBS-His	pMalc-5x	This study
	MBP-AUR	MBP-Aurein 1.2-CBS-His	pMalc-5x	This study
	MBP-GALA	MBP-GALA-CBS-His	pMalc-5x	This study
	MBP-LAH4	MBP-LAH4-CBS-His	pMalc-5x	This study
	MBP-HA2	MBP-HA2-CBS-His	pMalc-5x	This study
	Cas9-NLS	His-CBS-Cas9-NLS	pET19b	This study
	Cys-less Cas9-NLSx2	His-CBS-NLS-Cas9-NLS (C80S, C574S)	pET19b	This study

### Cell culture

BHK cells were maintained in a 37°C, 5% CO_2_ environment in growth media consisting of DMEM (GlutaMax, ThermoFisher) containing +4.5 g/L d-glucose and 1.9 mM Ca^2+^ with no sodium pyruvate) supplemented with 4 mM glutamine and 5% or 10% fetal bovine serum (FBS) as indicated. Cells were replated in coverslip slides in the same media 20–24 h before use in cell penetration assays.

### Cell penetration assays

Internalization assays were performed as described (Salerno et al., 2016; Ngwa et al., 2017) with minor modifications. Protein aliquots in a common storage buffer (10 mM HEPES, pH 7.4, 150 mM NaCl, 10% glycerol, 1 mM CaCl_2_) were thawed and sequentially assembled into complexes in this buffer on ice within 30 min of use. Following assembly, complexes were transferred to room temperature for 5–10 min and then diluted into media also at room temperature. After 1–3 min complex containing media was microcentrifuged at maximum velocity (>15,000 rcf) for 1 min to remove precipitates, briefly warmed in a 37°C bath (2–4 min) and transferred onto BHK cells from which growth media had just been removed. Unless otherwise stated, cells were treated with the indicated concentrations of complex at 37°C under 5% CO_2_ for 1 h, washed twice with PBS (containing Ca^2+^ and Mg^2+^) and analyzed in imaging media usually comprised of growth media without phenol red but including NucBlue, Live (ThermoFisher). Aliquots of proteins containing cysteine residues (Cas9-NLS and TAT-NMR-CaM) were thawed after addition of 1/20th volume of 1 mM DTT in storage buffer, which became less than 5 µM during cell treatment.

Internalization of TAT-CaM alone was assayed using DyLight 488 and 550 labeled adaptor modified with less than 0.3 dyes per protein. TAT-CaM in storage buffer was diluted 10-fold with imaging media and transferred onto BHK cells. TAT-CaM internalization of Cas9 used various labels on both proteins, as well as an optional treatment with green CMFDA CellTracker (Molecular Probes). When CellTracker was used, treatments were followed by a PBS wash and a 20 min incubation with PBS (mock) or CMFDA CellTracker in PBS, as indicated. TAT-CaM internalization experiments utilizing the “cysless” CBS-Tam were performed with various concentrations of an oligonucleotide (CCA​TCC​TGG​TCG​AGC​TGG​ACG​GCG​ACG) labeled with a 5′ Alexa 488 dye and a 3’ biotin equilibrated with CBS-Tam prior to complexation with the CPP-adaptor in Opti-MEM media (ThermoFisher), both preceded and followed by two washes with PBS and final addition of imaging media.

Confocal imaging was initiated as soon as practical (usually 7–15 min following the washes) and continued for up to 1 h in an incubated chamber that maintained a humidified, 37°C, 5% CO_2_ environment. In some experiments a parallel plate was initially treated identically; however, imaging was performed with the complex present between 20 and 60 min after addition. Cells with normal nuclei and monolayer distribution that avoided cell stacking were selected for imaging. Images were taken at a confocal plane just below the nuclear center to minimize slide surface background while retaining signal from intracellular structures across the cell. When NucBlue was not used, images were taken from a similar confocal plane above slide surface background that still included intracellular structures across the cell. Experiments used either a Zeiss LSM 700 or LSM 900 confocal microscope generally at 400× with settings adjusted to best image quality unless otherwise stated.

## Results and discussion

**FIGURE 1:**
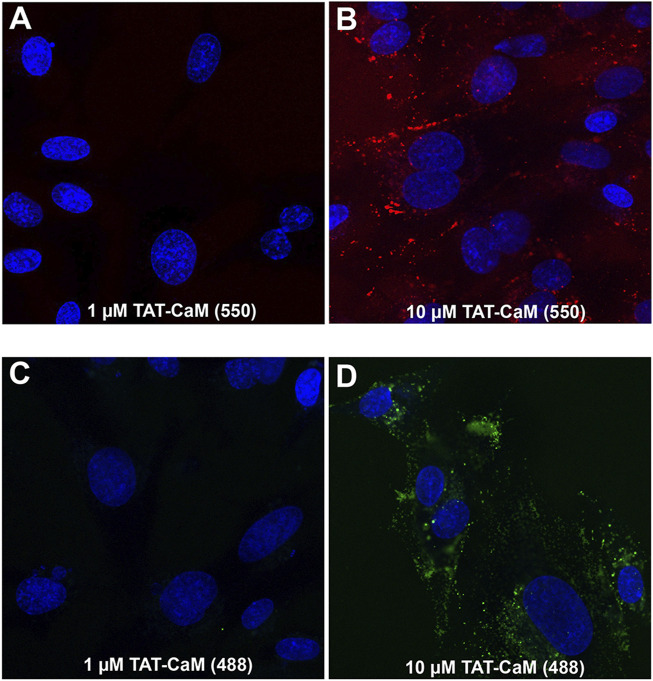
By itself, TAT-CaM internalization requires concentrations above 1 µM. BHK cells were incubated with **(A,B)** Dylight 550 or **(C,D)** Dylight 448 labeled TAT-CaM, respectively, for 60 or 30 min at 1 µM or 10 μM, in DMEM with 10% FBS under a 5% CO2 atmosphere at 37°C. Cells were then washed 3 times with PBS (with Mg^2+^ and Ca^2+^) and imaged in media without phenol red but containing NucBlue. Dye incorporation averaged less than 0.3 dyes per TAT-CaM molecule.

The CPP-adaptor technology was devised to facilitate dissociation of CPP and cargo, enabling cargo release from endosomally entrapped CPPs (LeCher et al., 2017). However, another facet of the CPP-adaptor technology is the simplicity of comparative analysis of adaptor, cargo and complex internalization properties. In this regard the properties of our CPP adaptor, TAT-CaM, are important for understanding TAT-CAM/cargo internalization. As observed for TAT-cargos and CPPs in general (Lönn and Dowdy, 2015), concentrations above 1 µM are necessary for effective internalization of TAT-CaM (Figure 1). By itself TAT-CaM is a rather small protein (∼26 kDa) whose properties may be altered by labeling; however, similar internalization was observed with modification by two amino-reactive fluorescent reagents, Dylight 550 (Figures 1A,B) and Dylight 488 (Figures 1C,D). To limit artifacts from labeling, TAT-CaM was modified at low efficiency (<0.3 dyes/molecule) with reduced concentrations of the reactive dyes below the manufacturer recommendations. Indeed, for the same reason, other proteins in this report were labeled at <0.6 dye/molecule to limit introduction of multiple dyes per protein monomer.

**FIGURE 2:**
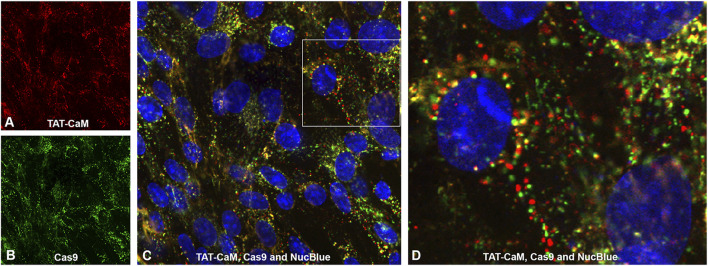
Internalization of Cas9 bound to TAT-CaM occurs readily at very low concentration. Cas9-NLS bound to equimolar TAT-CaM at 100 nM was incubated with BHK cells in DMEM with 10% FBS for 1 h prior to PBS treatment, washing and imaging. Panels show **(A)** 550-labeled TAT-CaM, **(B)** 488-labeled Cas9-NLS and **(C)** Composite including NucBlue. In **(D)** the enlarged inset (Figure 2D) shows Cas 9-NLS and TAT-CaM are often in separate but juxtaposed endosomes.

**FIGURE 3:**
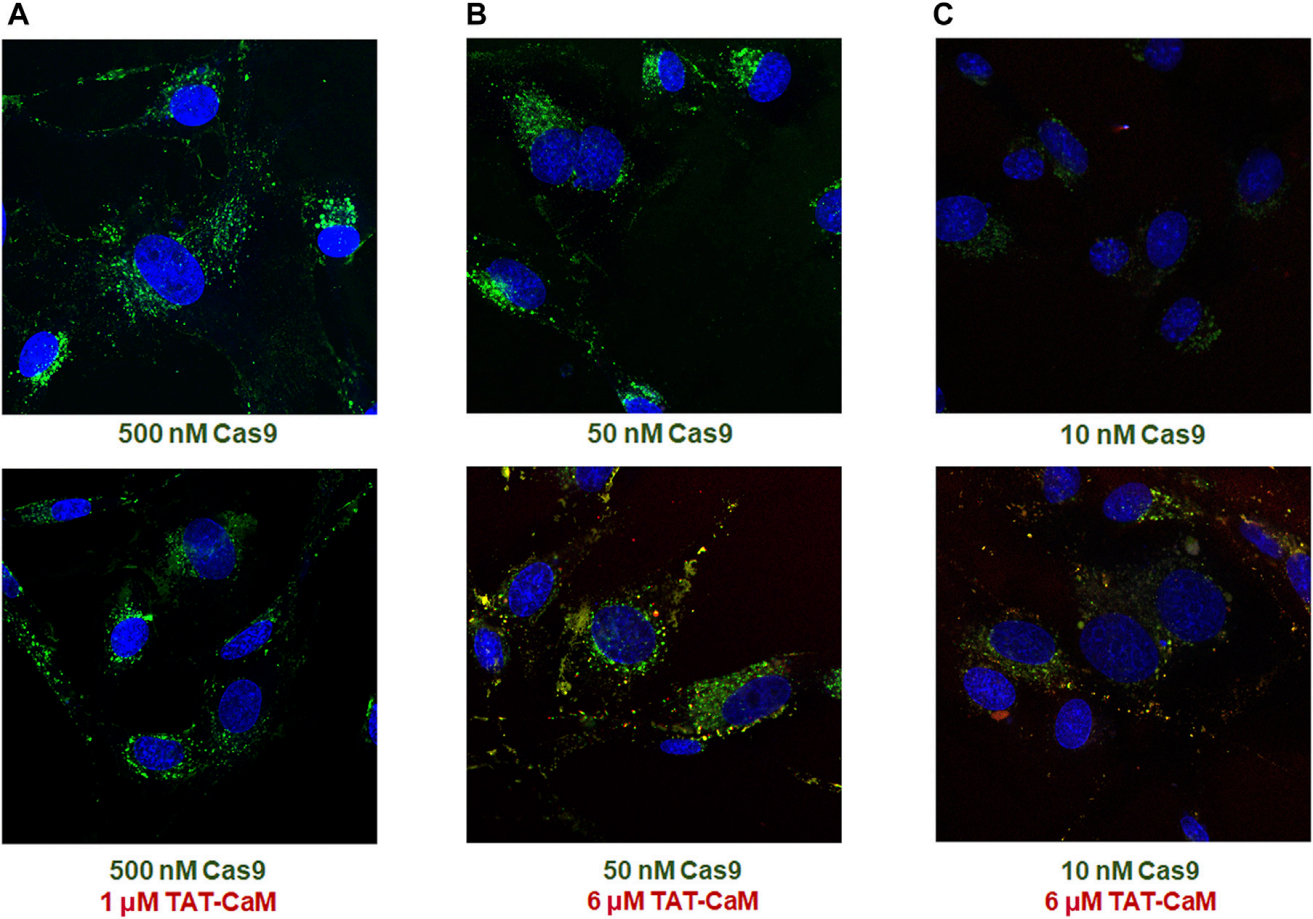
Specific and nonspecific Cas9 internalization with the TAT-CaM. BHK cells in DMEM with 10% FBS were treated for 1 h with Cas9-NLS in the absence (top) or presence (bottom) of excess TAT-CaM prior to washing (PBS x3) and imaging. Cas9-NLS at concentrations >50 nM showed no increase in internalization with inclusion of TAT-CaM as shown in examples **(A)** 500 nM and **(B)** 50 nM; however, at low Cas9-NLS concentrations including **(C)** 10 nM, TAT-CaM specifically increased internalization.

Unexpectedly, when labeled Cas9 (the protein component of CRISPR) containing a CBS tag and a nuclear localization signal (NLS) was internalized with equimolar 100nM TAT-CaM, the TAT-CaM/Cas9 complex internalized readily at a concentration that was more than 100-fold lower than observed for TAT-CaM alone (Figure 2). In other words, cargo internalization increased TAT-CaM internalization. Also note that TAT-CaM and Cas9 often appear separated but in nearby vesicular locations (Figure 2C and enlarged inset 2D). Importantly, under most conditions TAT-CaM does not increase Cas9 internalization, i.e. there is a lack of TAT-CaM dependent specificity (Figures 3A,B). On the other hand, at Cas9 concentrations below 50 nM where intrinsic internalization becomes modest, high TAT-CaM concentrations specifically increased Cas9 internalization (Figure 3C). The implication of this experiment is that Cas9 internalizes more readily when bound to TAT-CaM. However, this positive effect appears insignificant when intrinsic Cas9 internalization is high. In addition, we have observed other positively charged proteins used as cargos displayed similar intrinsic internalization (e.g. polyA binding protein, Gentry and McMurry, unpublished).

While at the time these results were mystifying, it has long been recognized that most CPPs are positively charged and may function by neutralizing negative charge on plasma membranes (Skotland et al., 2015), which is believed to aid inward membrane curvature necessary for vesicle internalization (Alves et al., 2010). Nevertheless, we were not aware of the extent to which surface charge of a cargo impacted CPP behavior as studies using covalently bound CPP moieties rarely test intrinsic cargo internalization ability.

It is also known that negative charge (Hymel et al., 2022) and nucleic acids (Yokoo et al., 2021) often inhibit CPP-mediated internalization. Concurrent with the Cas9 (CRISPR) experiments, a companion project involved development of an adaptor-based method to introduce donor DNA alongside CRISPR. To introduce biotin labeled nucleic acids, we created a CBS-tagged a version of avidin based on the expressible fungal homolog, tamavidin (Takakura et al., 2010; Takakura et al., 2013). With CBS-Tamavidin (Tam), biotin labelled nucleic acids can be delivered into cells as a Tam-oligo cargo complex reversibly bound to any adaptor. This version of “cysless” CBS-Tam (Tam) did not have an NLS and the lone cysteine was converted to serine to prevent disulfide-induced precipitation. As avidin orthologs including tamavidin form obligate tetramers (Takakura et al., 2009), as many as 4 nucleic acids can be bound to a single tetramer, which will have 4 CBS sites that can bind as many as 4 adaptors.

**FIGURE 4:**
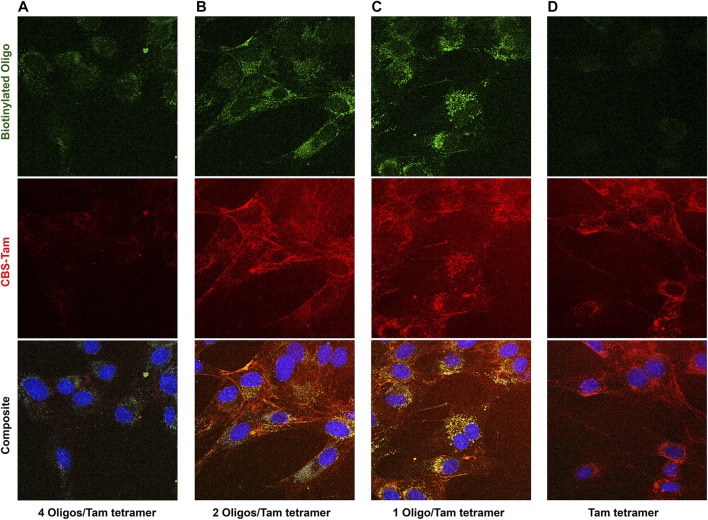
CBS-Tam, a cargo made from the fusion of CBS to the fungal avidin, tamavidin, internalizes model oligos but only at stoichiometries below 2 oligos per CBS-Tam tetramer. BHK cells were incubated with 600 nM TAT-CaM/Tam complexes bound to an oligo with a 5′ Alexa 488 dye and a 3′ biotin present at concentrations of **(A)** 600, **(B)** 300, **(C)** 150, or **(D)** 0 nM oligo for 1 h prior to washing (PBSx3) and imaging.

While early attempts to deliver labeled oligos clearly resulted in some internalization of the TAT-CaM/CBS-Tam/oligo complex, subsequent experiments showed CBS-Tam tetramers in the presence of a molar excess of oligos hardly enter the cell. This inconsistency was explained by decreasing the average number of oligos per tetramer, which produced the surprising result that decreasing oligo concentration increased oligo internalization (Figure 4). Maximal oligo internalization was observed at an average of 1 oligo per Tam tetramer (Figure 4C), although some entered at 2 oligos per Tam tetramer (Figure 4B). Beyond the nearly complete block to internalization, stoichiometric Tam/oligo concentrations also reduced complex binding to the glass coverslip. These results are consistent with increased negative charge decreasing the ability of the CPP to internalize cargo. While this explanation was accepted at the time, the dramatic inhibitory effect of oligo on complex internalization seems to indicate the oligo is forced into the proximity of the membrane and coverslip, suggesting steric hindrance may play a role.

**FIGURE 5:**
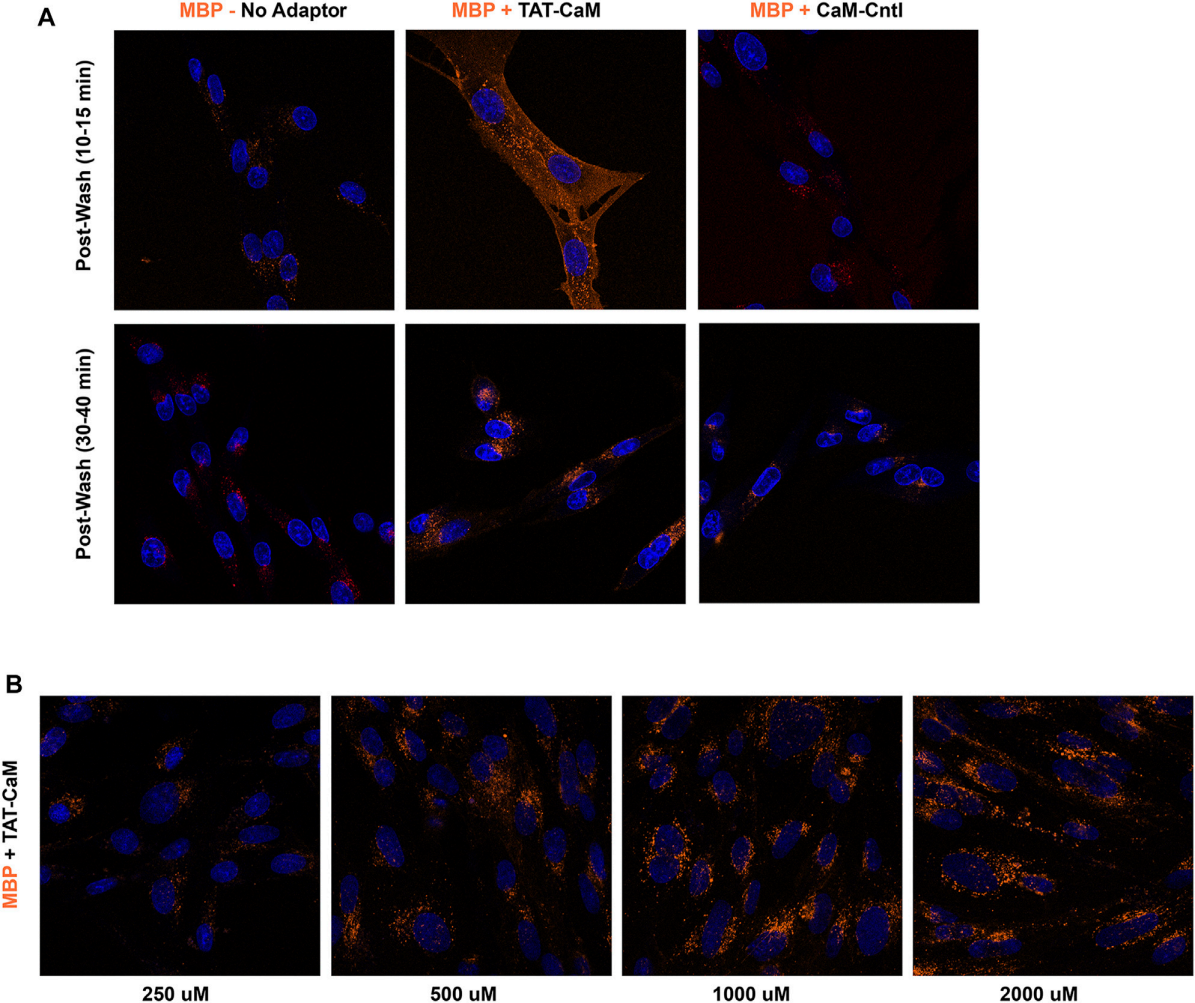
TAT-CaM specifically induces MBP-CBS internalization but requires µm concentrations. **(A)** Internalization of MBP-CBS alone (left panels), with equimolar with TAT-CaM (center panels) or with “TAT-less” CaM control (right panels) following 1 h incubation with BHK cells in growth media. Cells were washed (PBS x2) and imaged immediately (top panels) or later (bottom panels) in imaging media with NucBlue. **(B)** Internalization of equimolar MBP-CBS and TAT-CaM at 4 concentrations following a 2 h incubation, washing (PBS x2) and imaging in media with NucBlue.

To test the idea that a negatively charged cargo would display little intrinsic internalization, we created a CBS fusion cargo based on the negatively charged maltose binding protein (MBP). As predicted, MBP-CBS by itself displayed little tendency to enter BHK cells, but was induced to internalize when bound to TAT-CaM (Figure 5A). However, TAT-CaM-mediated internalization of MBP-CBS required a return to µM concentrations (Figure 5B) characteristic of typical CPP-mediated internalization processes. To test the extent to which the negative charge of CaM was inhibitory, the internalization of a TAT-less CaM control (CaM-Cntl) in complex with MBP was concurrently examined and found to further reduce MBP-CBS internalization (Figure 5A, left panel and Figures 8–10 below). Importantly, comparison of TAT-CaM/MBP-CBS to (TAT-less) CaM-Cntl/MBP-CBS isolates the effect of the TAT sequence on complex internalization, a conclusion that is valid because neither CaM nor MBP contribute significantly to the internalization process.

**FIGURE 6:**
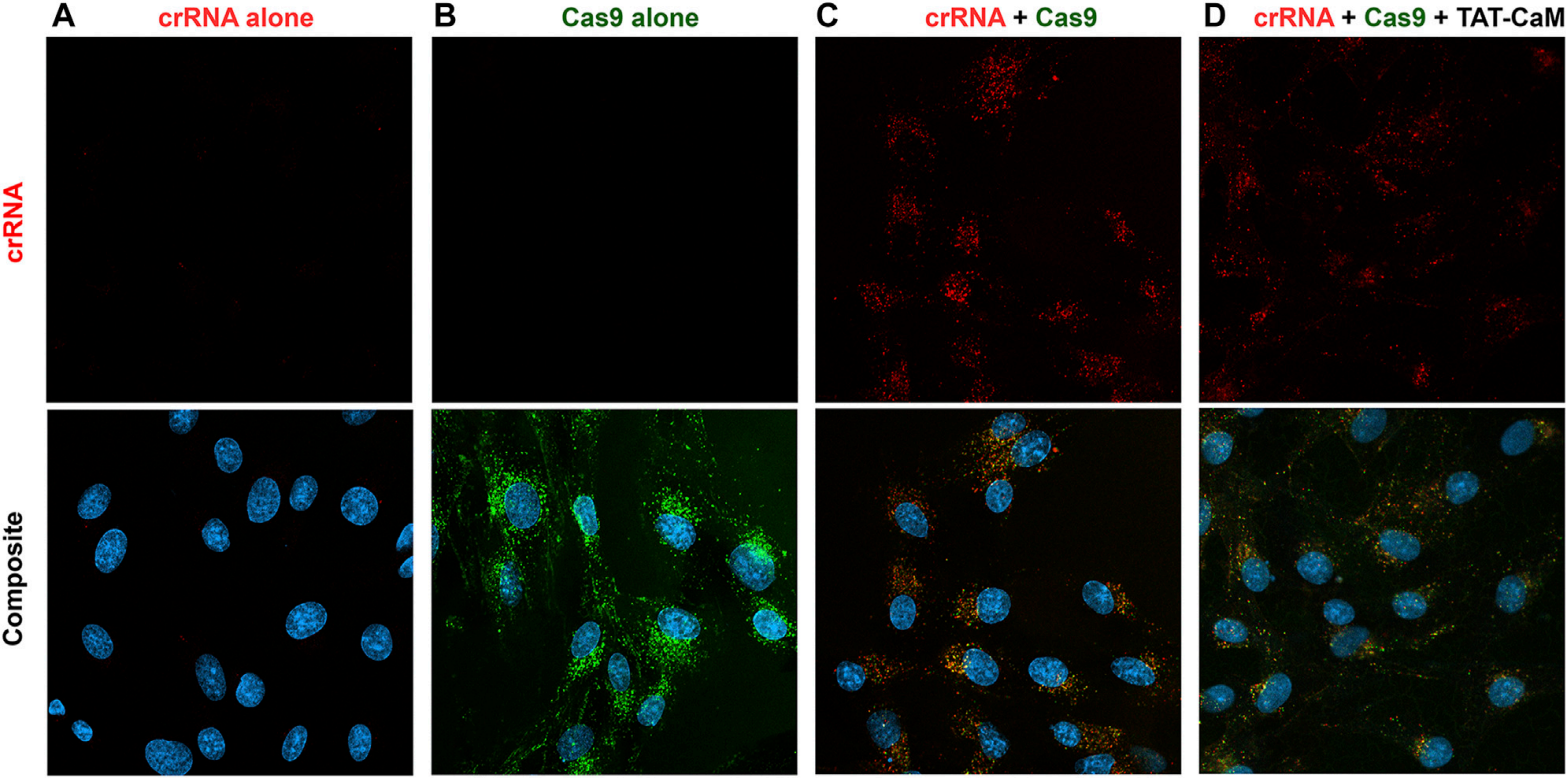
Internalization of CRISPR components and CRISPR compared to the TAT-CaM/CRISPR complex. BHK cells in DMEM with 5% FBS were treated for 1 h with **(A)** crRNA, **(B)** Cys-less-Cas9-NLSx2, **(C)** CRISPR or **(D)** the TAT-CaM/CRISPR prior to washing (PBS x2) and imaging in media with NucBlue. The Cys-less Cas9-NLSx2 concentrations were 100 nM; however, the crRNA and TAT-CaM were used at 150 nM to ensure available Cas9 was fully bound.

Given these results, it might be expected that association of a crRNA with Cas9 would decrease intrinsic internalization. Indeed, internalization of “cysless” CBS-Cas9-NLSx2 (Figure 6B) was much greater than observed when it was part of the CRISPR complex (Figure 6C). Nevertheless, the CRISPR complex still internalized readily (Figure 6C) and TAT-CaM still had no ability to detectably increase CRISPR internalization and in some experiments seemed to be somewhat inhibitory (Figure 6D). Because the ATTO 550 crRNA duplex displays virtually no cell penetration by itself (Figure 6A), essentially all crRNA inside the cell entered as part of the CRISPR complex. Thus, the crRNA provides a measure of CRISPR internalization. When Cas9 was not labeled similar amounts of crRNA/Cas9 internalization were observed (unpublished, D. Morris). Although TAT-CaM failed to increase CRISPR internalization, intrinsic internalization and frequent TAT-CaM-dependent effects on the details of intracellular localization (Figures 6C,D) suggested some potential for genome modification. Unfortunately, even with CRISPR complexes that both cleaved target DNA *in vitro* and modified genomic DNA when introduced using CRISPR-Max, no detectable genome modification occurred when CRISPR was introduced with or without TAT-CaM in HEK-293 (Supplementary Figure S2) or BHK cells (Morris and McMurry, unpublished).

The strategy of using noncovalent association of adaptor and cargo allowed independent modification of adaptors and cargos to increase internalization. Further, combinatorial inclusion of the Endolytic Peptides (EPs) within both adaptors and cargos should increase endosomal escape and aid internalization as the beneficial properties of these peptides may be due to both factors. To explore these possibilities, we chose a set of 4 EPs, Aurein 1.2 (AUR), GALA, LAH4 and HA2, previously suggested to increase endosomal escape, and incorporated these sequences into a set of MBP-EP-CBS cargos and TAT-EP-CaM adaptors. Problematically, association of negative TAT-CaM with negative MBP produces complexes with modest internalization, which probably limits endolytic outcomes, nevertheless, these modified adaptors and cargos produced clear and differential effects on complex internalization.

**FIGURE 7:**
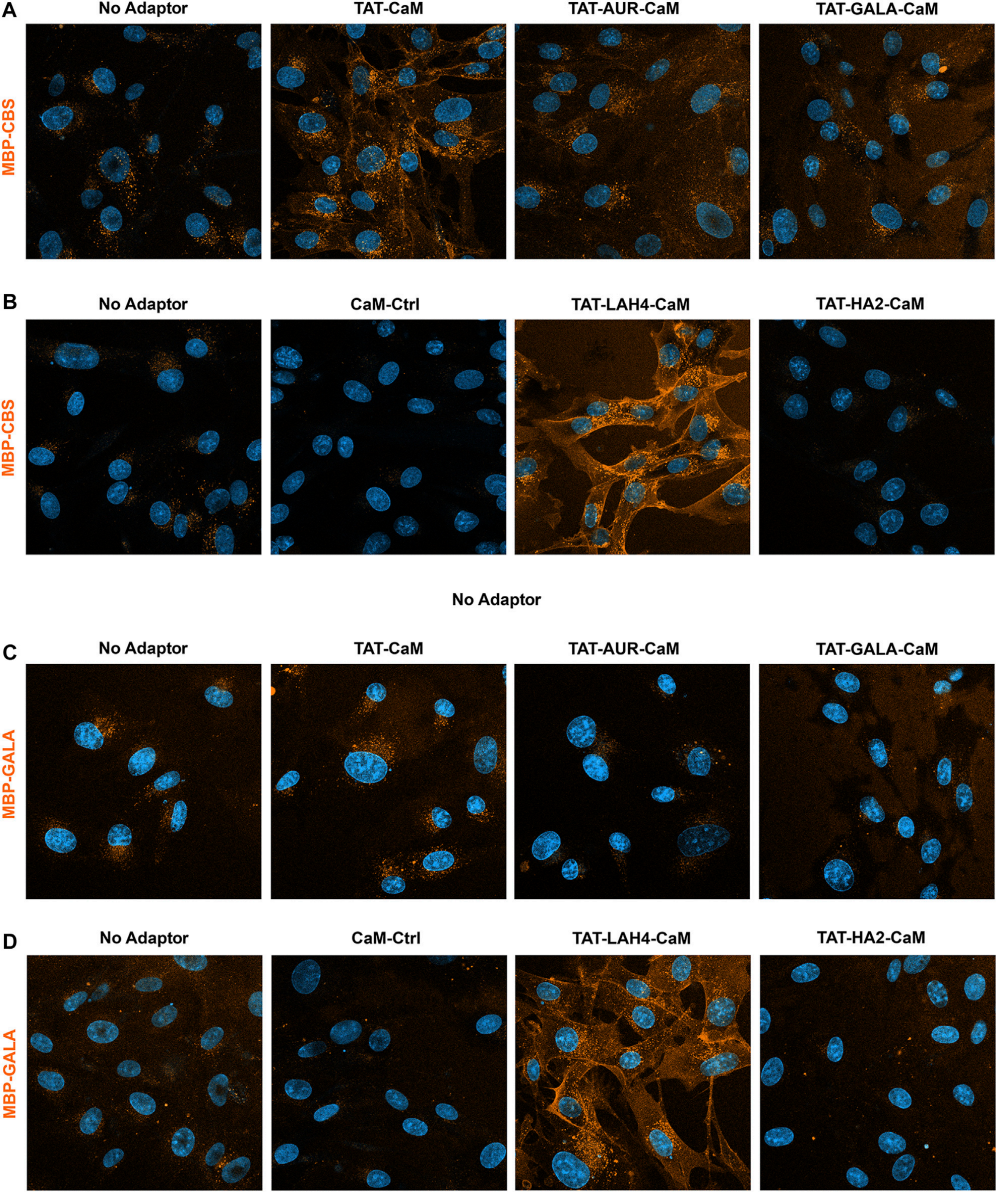
550- MBP-CBS and 550- MBP-GALA internalized with adaptor set including controls. Internalization of equimolar adaptor/cargo complexes (1 µM) in growth media were analyzed as sets of four conditions, each containing the no adaptor cargo as reference. All complexes were internalized for 1 h prior to washing (PBS x2) and imaging in media containing NucBlue. Internalization of CBS-MBP in the upper panel **(A)** used: no adaptor, TAT-CaM, TAT-AUR-CaM, or TAT-GALA-C and in the lower panel **(B)** used: no adaptor, “TAT-less” CaM-Ctrl, TAT-LAH4-CaM, and TAT-HA2-CaM. Internalization of MBP-GALA used the same adaptor sets in the upper **(C)** and lower **(D)** panels. For presentation, intensities within each set were adjusted identically: however, for these cargos no-adaptor reference are not presented at equal intensity in upper and lower panels to avoid oversaturating internalization with TAT-LAH4-CaM.

Initially, intrinsic internalization of each cargo alone was compared to that observed with TAT-CaM, four TAT-EP-CaMs and CaM-Cntl to obtain a profile of adaptor effectiveness. To limit the time differential between imaging treatments, experiments were performed in sets of 4, each containing the no adaptor control as a reference. Multiple rounds of images were taken as soon as possible after complex removal and PBS washing. As shown above, relative to MBP-CBS alone, association with TAT-CaM specifically increased internalization (Figure 7A). Internalization of MBP-CBS by TAT-LAH4-CaM was also very efficient (Figure 7B), while TAT-AUR-CaM was marginally effective (Figure 7A) and CaM-Cntl depressed internalization (Figure 7B).

With MBP-GALA as cargo, the adaptors displayed similar activity as with MBP-CBS. Compared to MBP-GALA alone, TAT-CaM modestly increased internalization (Figure 7C), while TAT-LAH4-CaM remained a very effective adaptor (Figure 7D) and CaM-Cntl was again inhibitory (Figure 7D).

**FIGURE 8:**
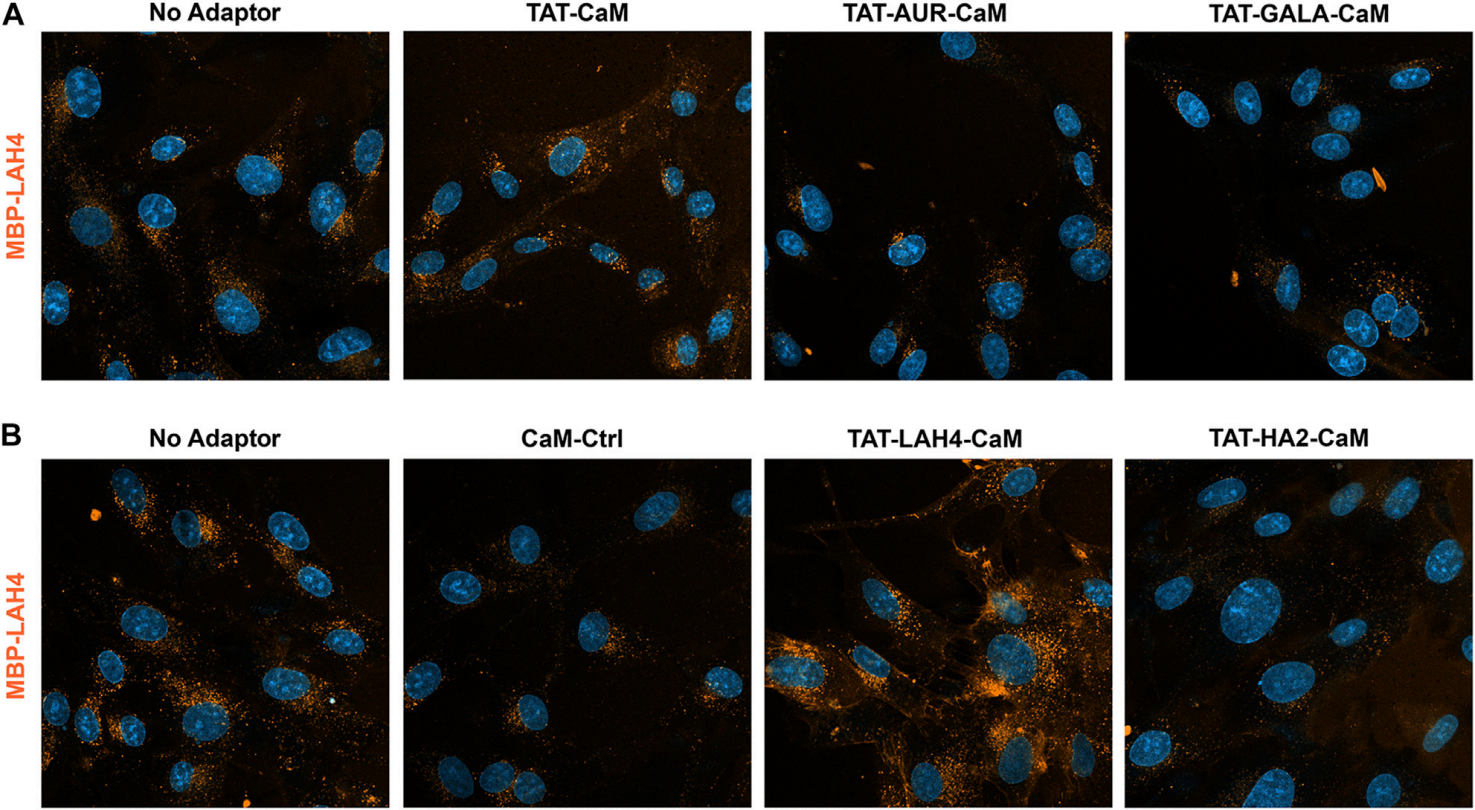
550-MBP-LAH4 internalized with adaptor set including controls as in the prior figure. Equimolar adaptor/cargo complexes (1 µM) were internalized in growth media for 1 h prior to washing (PBS x2) and imaging in media containing NucBlue. Internalization of MBP-LAH4 with adaptors as above in both the upper panel **(A)** and the lower panel **(B)**. For presentation, intensities within each set adjusted identically but with the no-adaptor reference at approximately equal intensity for both panels.

The MBP-LAH4 cargo displayed strong internalization across the adaptor set (Figure 8) and reduced detection sensitivity was required to produce non-saturating signal. As shown more directly below, the LAH4 sequence in MBP-LAH4 itself acted as an effective CPP. Against this background of higher internalization, TAT-CaM increased MBP-LAH4 internalization modestly (Figure 8A) while TAT-LAH4-CaM was still very effective (Figure 8B). Unexpectedly, TAT-AUR-CaM, TAT-GALA-CaM and TAT-HA2-CaM were inhibitory and decreased MBP-LAH4 internalization much as CaM-Cntl.

**FIGURE 9:**
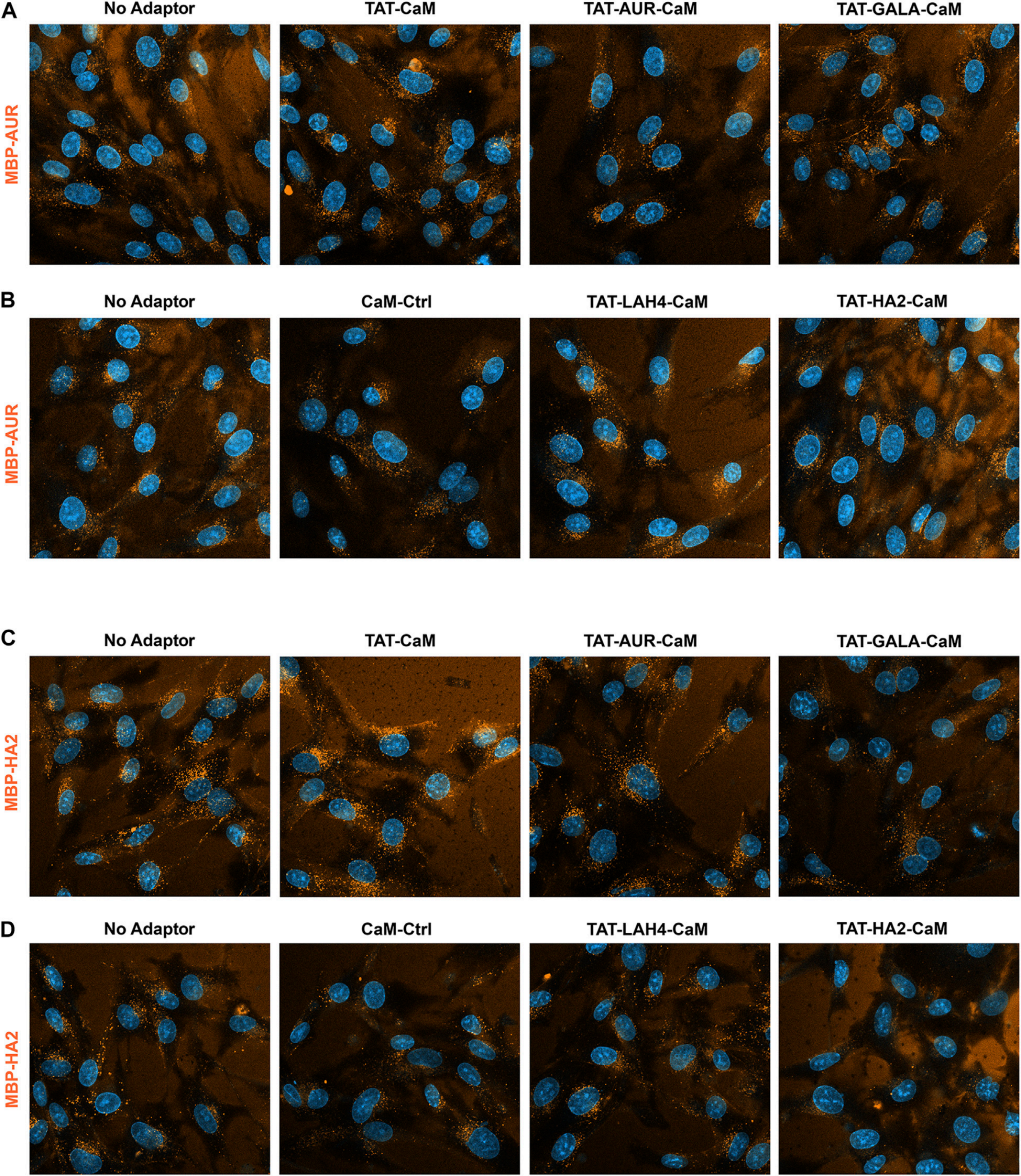
550-MBP-AUR and 550-MBP-HA2 internalized with adaptor set including controls as in prior 2 figures. Equimolar adaptor/cargo complexes (1 µM) were internalized in growth media for 1 h prior to washing (PBSx2) and imaging in media containing NucBlue. Internalization of MBP-AUR and MBP-HA2 with the adaptors as above in both the upper panels **(A,C)** and the lower panels **(B,D)**. For presentation, intensities within each set adjusted identically but with the no-adaptor reference at approximately equal intensity for all panels.

Cargos MBP-AUR and MBP-HA2 displayed similar but distinctly alternative behaviors (Figure 9). Both EPs appeared to have some CPP capability of their own (see below) but also bound the coverslip surface complicating analysis. In addition, both cargos were resistant to increased internalization by other adaptors, although TAT-CaM showed some efficacy. Most strikingly, TAT-LAH-CaM had modest impact on MBP-AUR (Figure 9B) and no impact on MBP-HA2 internalization despite this adaptor’s effectiveness with the other cargos (Figure 9D). Also noteworthy was the loss of MBP-HA2 internalization when complexed with TAT-HA2-CaM to levels below that of CaM control (Figure 9D).

Although too large for presentation in the body of this report, the overall internalization characteristics of the MBP-EP-CBS cargos and TAT-EP-CaM adaptors are most apparent in a noncolor matrix simultaneously displaying all the representative images (Supplementary Figure S3). The reproducibility of the pattern is shown in a nearly complete second matrix performed at half concentration (0.5 µM) that is missing only the CaM control (Supplementary Figure S4). Significantly, both data sets demonstrate the ability of TAT-LAH4-CaM to dramatically increase internalization of MBP-CBS, MBP-GALA and MBP-LAH4, while having minimal efficacy with MBP-AUR and MBP-HA2. Further, the reciprocal exchange of LAH4 (into cargo) and Aurein and HA2 (into adaptor) produced poor internalization in both experiments, suggesting this negative interaction is independent of context. More broadly, the comparison of adaptor effectiveness confirmed the utility of the TAT and LAH4 sequences and demonstrated that inclusion of GALA, AUR and HA2 sequences in the adaptor did not increase internalization.

**FIGURE 10:**
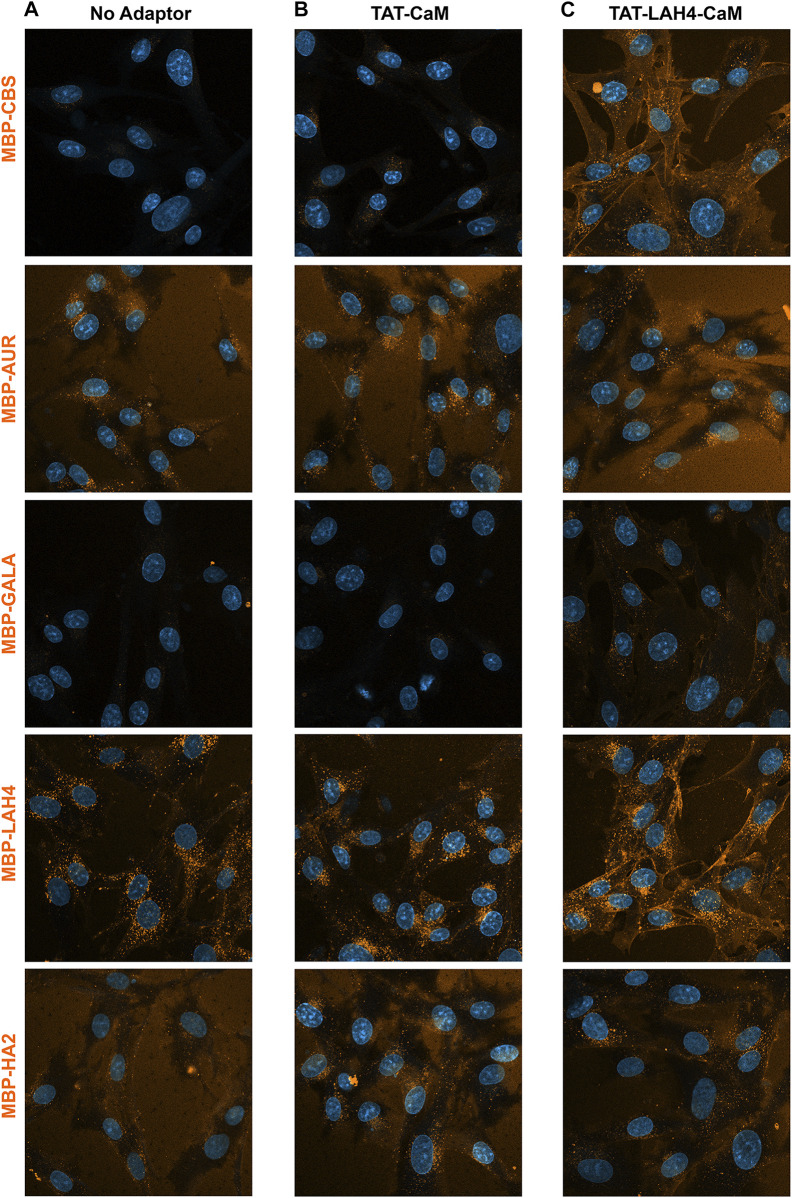
Direct comparison of internalization efficiencies for five 550-labeled MBP-EP-CBS cargos: MBP-CBS, MBP-AUR, MBP-GALA, MBP-LAH4, and MBP-HA2. Cargo alone and adaptor/cargo complexes (1 µM) were internalized in growth media for 1 h prior to washing (PBS x2) and imaging in media containing NucBlue. Internalization of the 5 substrates was concurrently analyzed in **(A)** with no adaptor present, in **(B)** with TAT-CaM and in **(C)** with TAT-LAH4-CaM. The individual experiments represented by each column were independently adjusted to accent pattern differences within each column (for direct comparison of adaptors see Figures 7–9).

While beneficial EP effects on cargo internalization are implied by reduced sensitivity used in detection, direct comparison provided a more quantitative measure. To compare these EP effects, all 5 cargos were simultaneously analyzed first alone and then with selected adaptors. Comparison of the intrinsic internalization tendencies of the EP-cargos showed LAH4-MBP internalized readily, MBP-AUR and MBP-HA2 to some extent and MBP-CBS and MBP-GALA very little (Figure 10A). Stated alternatively, when fused into an MBP-CBS cargo, LAH4 is a relatively good CPP, Aurein, and HA2 have CPP characteristics and GALA appears to have no such property.

As shown above, TAT-CaM has some ability to increase internalization of all 5 cargos. Nevertheless, internalization of MBP-EP-CBSs by TAT-CaM (Figure 10B) correlated with the intrinsic internalization observed for the cargos themselves. Indeed, internalization of the adaptor/cargo complexes maintained almost the same rank order as observed for the cargos alone (MBP-LAH4>MBP-AUR > MBP-HA2>MBP-CBS = MBP-GALA) except that TAT-CaM-induced internalization of MBP-CBS became visible (MBP-CBS > MBP-GALA). These results showed that substrate EPs still played a major role in boosting the internalization process, but the overall efficiency was dependent on both cargo EPs and TAT-CaM.

On the other hand, when the TAT-LAH4-CaM was used as an adaptor, the internalization properties of the complexes changed qualitatively (MBP-LAH4>MBP-CBS > MBP-AUR > MBP-HA2 = MBP-GALA) (Figure 10C). While MBP-GALA internalization remained low, the relative signal became similar to HA2-MBP, in agreement with above data showing TAT-LAH4-CaM adaptor dramatically increased internalization of MBP-GALA (Figure 7D). These data show that internalization stimulated by the adaptor was more significant to the internalization process than the CPP effects of the cargo-EP peptides.

Given the beneficial role of positive charge during the internalization process and recognition that the CaM portion of TAT-CaM was acidic and inhibited internalization, it was clear that more effective adaptors were needed. Several approaches were used to increase the internalization potential of these next-generation adaptors and one of the most successful involved use of CaM from naked mole rat to create a TAT-NMR-CaM adaptor (Gentry et al., 2021). A second approach used an engineered positive version of GFP (pGFP), which has been shown (Lawrence et al., 2007; Thompson et al., 2012) to be a powerful cell penetrating protein (also ‘CPP’), to create a pGFP-CaM adaptor. Although full characterization of these very different adaptors is beyond the scope of this report, it seemed valuable to see how these more effective adaptors interacted with the MBP-EP-CBS cargos.

**FIGURE 11:**
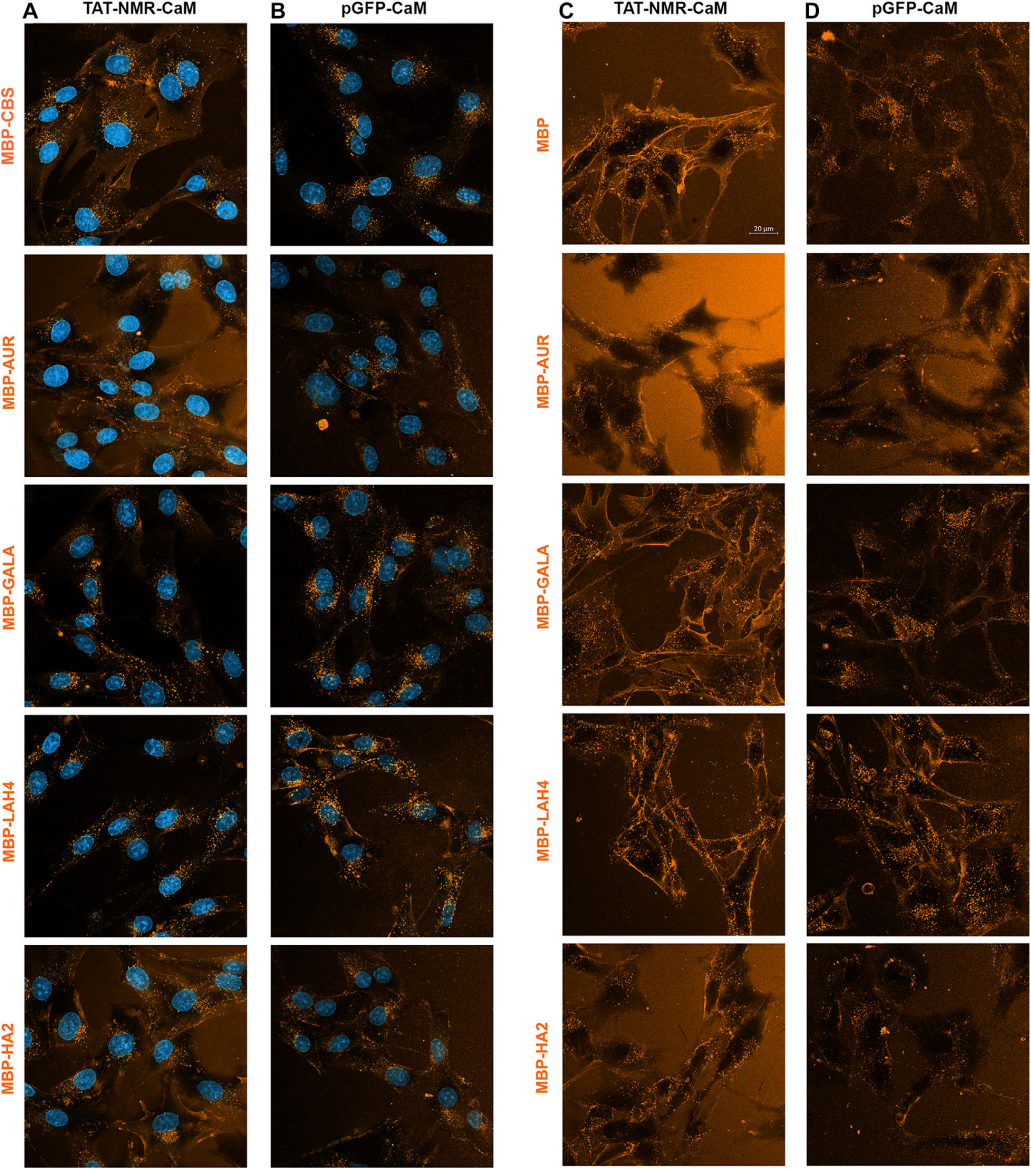
Direct Comparison of internalization efficiencies for the MBP-EP-CBS cargos internalized by two next generation adaptors. **(A)** Internalization of the five 550-labeled MBP-EP-CBS cargos by TAT-NMR-CaM **(A)** and pGFP-CaM **(B)** under the same conditions used with TAT-EP-CaM adapters. Internalization of these 5 cargos with **(C)** TAT-NMR-CaM and **(D)** pGFP-CaM analyzed in the later part of a 1 h internalization incubation when the complex is present in solution.

When TAT-NMR-CaM was used to internalize the EP-cargo set, cargos having the least intrinsic internalization ability (MBP-CBS and MBP-GALA) were induced relative to those with strong intrinsic internalization (Figure 11A). While all cargos internalized similarly, the cargo without an EP (MBP-CBS) internalized most readily. MBP-AUR internalized the least when non-background signal was considered. Clearly with the TAT-NMR-CaM adaptor, specific internalization had become robust enough that intrinsic internalization was no longer a major factor.

When pGFP-CaM was used to internalize the EP-cargo set, MBP-CBS and MBP-GALA joined MBP-LAH4 as the cargos that internalized most readily (Figure 11B). If adaptor efficacy were the only factor driving internalization, then MBP-AUR and MBP-HA2 should have increased as well. However, relative internalization was clearly lower.

Additional evidence more dramatically demonstrated the unusual behavior of the MBP-AUR and MBP-HA2 cargos. When EP-cargo internalization by TAT-NMR-CaM (Figure 11C) and pGFP-CaM (Figure 11D) was analyzed with adaptor/cargo complexes still present, internalization patterns of MBP-AUR and MBP-HA2 were strikingly different from other cargos. Particularly for MBP-AUR, little intracellular cargo was apparent and there was a black space in the cell center. Indeed, the lack of intracellular MBP-AUR internalized by pGFP-CaM during treatment (Figure 11D) did not appear consistent with that present after washing (Figure 11B) in these concurrent experiments, suggesting a wave of internalization associated with the washing event.

**FIGURE 12:**
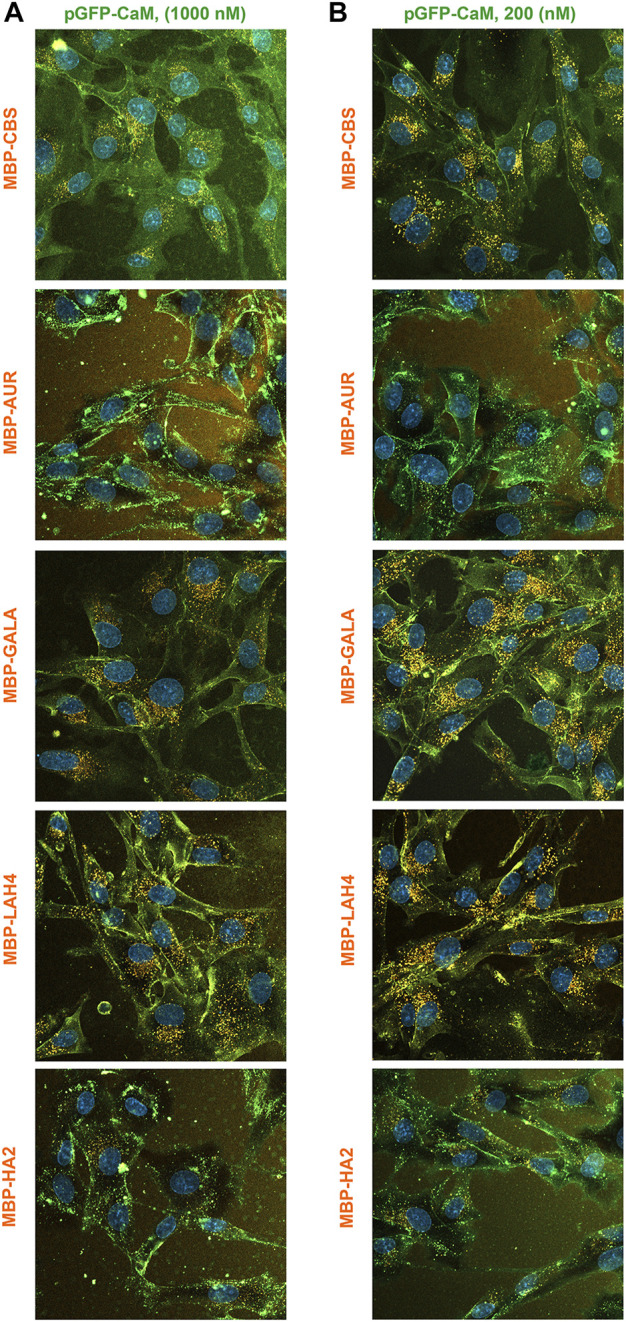
Colocalization of MBP-EP-CBS cargos and pGFP-CaM during comparison of the internalization of the five MBP-EP-CBS cargos at two concentrations. Simultaneous internalization of the five 550-labeled MBP-EP-CBS cargos with equimolar pGFP-CaM at complex concentrations of **(A)** 1000 nM or **(B)** 200 nM. Equimolar adaptor/cargo complexes were internalized in growth media containing NucBlue and imaged with the complex present between 40–60 min.

In contrast to the other adaptors, the fluorescence of the pGFP-CaM allowed simultaneous analysis of adaptor and cargo internalization. When imaged with adaptor/cargo complexes present at a concentration of 1 μM, colocalization of pGFP-CaM and cargos again broke into two groups with MBP-AUR and MBP-HA2 producing much lower levels of internalized cargo apparent as green cells (Figure 12A). The cell surface staining observed with MBP-CBS is a characteristic of this cargo that occurred at high complex concentration, a situation that also appeared to produce a wave of internalization associated with the washing event. However, when the concentration of pGFP-CaM/cargo complexes was reduced 5-fold (Figure 12B), the MBP-CBS pattern both increased in intensity and assumed a similar appearance to that of MBP-GALA and MBP-LAH4. In contrast, both MBP-AUR and MBP-HA2 retained their distinctive green appearance, although modest internalization of pGFP-CaM/MBP-AUR became more apparent. Nevertheless, the relative ineffectiveness of both EP and next generation adaptors at inducing MBP-AUR and MBP-HA2 internalization strongly suggests that intrinsic properties of the EP sequences are responsible for the differences.

## Conclusion

The primary conclusion of these studies is that cargo characteristics often play an important role in CPP associated internalization. Further, net charge may be a dominating characteristic governing CPP-adaptor mediated internalization efficiency. Our recent experience suggests that cargo-induced increases in complex internalization represent the typical behavior observed with cargo proteins having a net charge ranging from positive to neutral. A dramatic example of the ability of charge to increase intrinsic cargo internalization can be seen in supercharged GFP, which was created by mutating surface residues to R or K and produced a powerful cell penetrating protein capable of delivering a covalently attached cargo (Lawrence et al., 2007; McNaughton et al., 2009). This supercharged GFP was of course used in the design of pGFP-CaM and produced an adaptor that appears to be a powerful CPP with most cargos.

The experiments presented here represent a fraction of the conditions, cargos and methodologies used during our efforts to understand why some cargos including Cas9 displayed no TAT-CaM-specific increase in internalization. While the ability of positive charge to enable CPP activity is well established, the dominating effect cargo charge can have on this process seems to be an understudied topic. Between that space where the CPP dominates the internalization process and where the adaptor has marginal impact on strong intrinsic cargo internalization may lie the general case where both adaptor (CPP) and cargo are positively impacting membrane association and internalization. This is not particularly surprising and represents a specific case of what has been termed the A-B site problem (Jencks, 1981). When two binding sites can independently associate with a target, two relatively weak sites can generate binding beyond that expected from addition of their individual free energy because the first binding event pays much of the entropic price of association, increasing the enthalpic benefit of the second site association. Clearly, cargo characteristics including charge seriously complicate investigation of CPP-adaptor function and behavior. This is not a flaw in the adaptor concept but rather a characteristic of CPP/cargo internalization events. Indeed, a CPP-less control cargo is an important control even for fused CPP-cargo proteins. Clearly, cargo-induced internalization complicates interpretation of the existing CPP literature based on covalent association of CPPs and cargos.

There are practical considerations that are unique to and essential for the interpretability of CPP-adaptor/CBS-cargo experiments. In general, the primary goal of CPP internalization is to increase cargo entry. However, when most of the basis of cargo entry resides in characteristics of the cargo (e.g., positive charge), unexpected outcomes can occur. These include not only an inability of the adaptor to increase adaptor/cargo internalization, but even adaptor inhibition of the internalization process. When the adaptor inhibits complex internalization, as occurred in some situations with TAT-CaM, the adaptor was used in excess of cargo, so that all internalizing cargo was bound by the inhibitory adaptor. Of course, the reciprocal is also the case, when the adaptor drives internalization of inhibitory cargo, the cargo needs to be in excess for interpretation of adaptor internalization to be interpretable. These factors were a consideration during CRISPR internalization and an excess of both the crRNA duplex and TAT-CaM were used, so that essentially all Cas9 entering the cell did so as part of a complete complex. The ability of the MBP-AUR cargo to block pGFP-CaM internalization suggests pGFP-CaM was not in excess during that experiment. As noted, the amount of MBP-AUR apparent inside washed cells was not consistent with the nearly complete block of MBP-AUR and pGFP-CaM internalization apparent in images taken while the complex was present in the medium. This suggests that a wave of internalization accompanies washing, a phenomenon that appears fairly common. We have avoided experiments, usually involving high complex concentrations, where adaptor/cargo complexes strongly display this behavior because this issue is beyond the scope of the current study. Significantly, a reduction in pGFP-CaM/MBP-AUR concentration increased internalization during the incubation period. However, for this cargo, inhibition of entry remains readily apparent.

The TAT-CaM adaptor was obviously not designed based on the fact that CaM was a very negative protein that could isolate CPP sequence efficacy. As the effect the negative CaM was having on internalization became apparent (see data on the TAT-less CaM control), this characteristic was regarded as an unequivocal negative. However, recognition that CPP-EP-CaM fusions provided adaptors whose internalization ability was based almost entirely on the CPP characteristics has proved valuable. In combination with cargos based on intrinsically negative MBP, which also provides little propensity for internalization, complexes can be created in which CPP and EP sequences can be combinatorially tested to improve internalization and probably cargo escape. In this study, we focused entirely on effects the 4 EP sequences had on internalization because it was amenable to confocal analysis. The surprising benefits of the LAH4 as a CPP, apparent in both the TAT-LAH4-CaM adaptor and the LAH-MBP cargo, suggest the sequence will have general utility in stimulating cell penetration. Clearly, adding the LAH4 sequence to TAT-NMR-CaM and GFP-CaM provides one potential avenue for establishing the utility of this sequence in creating even more powerful adaptors.

The negative consequences the AUR and HA2 sequences on cargo internalization may not recommend their use as CPPs but does not preclude their utility as EPs with the right CPP-adaptor. Indeed, given the striking phenotype associated with inclusion of these relatively short sequences in the MBP cargos, it is tempting to speculate that their previously identified EP properties are responsible. Because the TAT-EP-CaM/MBP-EP model produced relatively low internalization and because endosomal escape is not easily captured with confocal microscopy, investigation of combinatorial endolytic properties of EP sequences in adaptors and cargos has not been addressed here. Future experiments designed to measure endosomal escape using EPs in the adaptor context will require incorporation of a reporter that directly measures escape, e.g. (Milech et al., 2015; Teo et al., 2021). While the ability of the TAT-EP-CaM/MBP-EP system to measure endosomal escape remains to be established, our adaptor/cargo strategy clearly has utility in investigating the roles of CPP and EP sequences in the internalization process. This system may prove quite valuable in pursuit of even more effective CPPs that can deliver large macromolecular cargoes with all the therapeutic potential implicit in CPP capability.

## Data Availability

The datasets presented in this study can be found in online repositories. The names of the repository/repositories and accession number(s) can be found in the article/Supplementary Material.
