# Erratum: A reversible cell penetrating peptide-cargo linkage allows dissection of cell penetrating peptide- and cargo-dependent effects on internalization and identifies new functionalities of putative endolytic peptides

**DOI:** 10.3389/fphar.2023.1201245

**Published:** 2023-04-18

**Authors:** Frontiers Production Office

**Affiliations:** Frontiers Media SA, Lausanne, Switzerland

**Keywords:** cell-penetrating peptides, protein transduction domains (PTD), endolytic peptides, endocytosis, calmodulin (CAM)

Due to a production error, several units throughout the article were incorrectly formatted (“mm” instead of “mM”, “nm” instead of “nM”, “µm” instead of “µM”, and “pm” instead of “pM”).

The publisher apologizes for this mistake. The original version of this article has been updated.

