# Corrigendum: A reversible cell penetrating peptide-cargo linkage allows dissection of cell penetrating peptide-and cargo- dependent effects on internalization and identifies new functionalities of putative endolytic peptides

**DOI:** 10.3389/fphar.2023.1152506

**Published:** 2023-04-18

**Authors:** Daniel P. Morris, Lucy C. Snipes, Stephanie A. Hill, Michael M. Woods, Maria M. Mbugua, Lydia R. Wade, Jonathan L. McMurry

**Affiliations:** Department of Molecular and Cellular Biology, Kennesaw State University, Kennesaw, GA, United States

**Keywords:** cell-penetrating peptides, protein transduction domains (PTD), endolytic peptides, endocytosis, calmodulin (CAM)

In the published article, there was an error in the legend for **Figure 12** as published.

[As in the prior 5 figures, equimolar adaptor/cargo complexes were internalized in growth media for 1 h prior to washing (PBS x2) and imaging in media containing NucBlue.]. The corrected legend appears below.

“Colocalization of MBP-EP-CBS cargos and pGFP-CaM during comparison of the internalization of the five MBP-EP-CBS cargos at two concentrations. Simultaneous internalization of the five 550-labeled MBP-EP-CBS cargos with equimolar pGFPCaM at complex concentrations of (A) 1000 nM or (B) 200 nM. Equimolar adaptor/cargo complexes were internalized in growth media containing NucBlue and imaged with the complex present between 40–60 min.”

The authors apologize for this error and state that this does not change the scientific conclusions of the article in any way. The original article has been updated.

