# Supplementary figures and images for: A reversible cell penetrating peptide-cargo linkage allows dissection of cell penetrating peptide- and cargo-dependent effects on internalization and identifies new functionalities of putative endolytic peptides
Source: Front Pharmacol. 2022 Nov 21;13:1070464. doi: 10.3389/fphar.2022.1070464 (PMC9720253; doi:10.3389/fphar.2022.1070464)

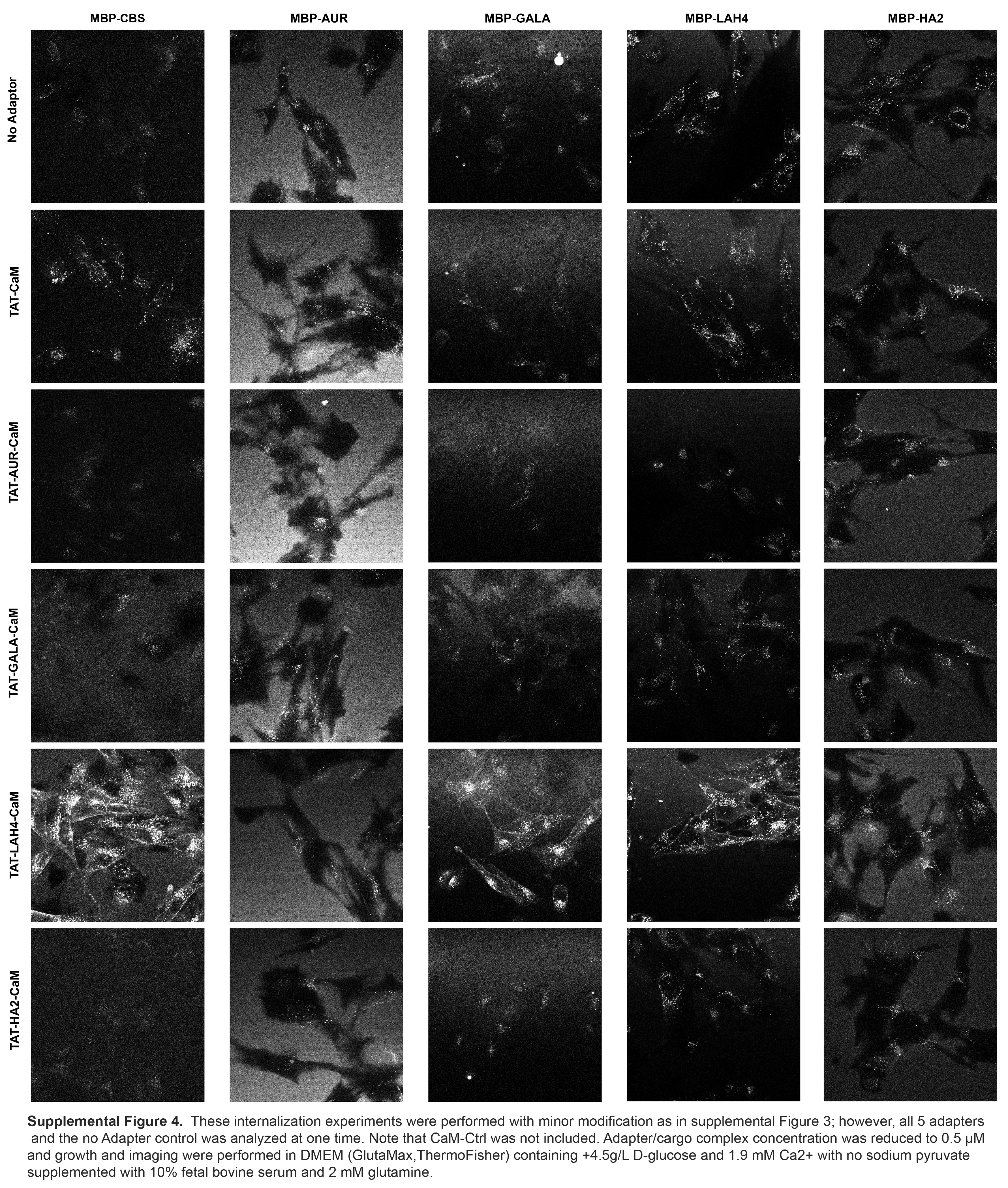

Supplement: Supplementary file 1 [file Image3.TIF]

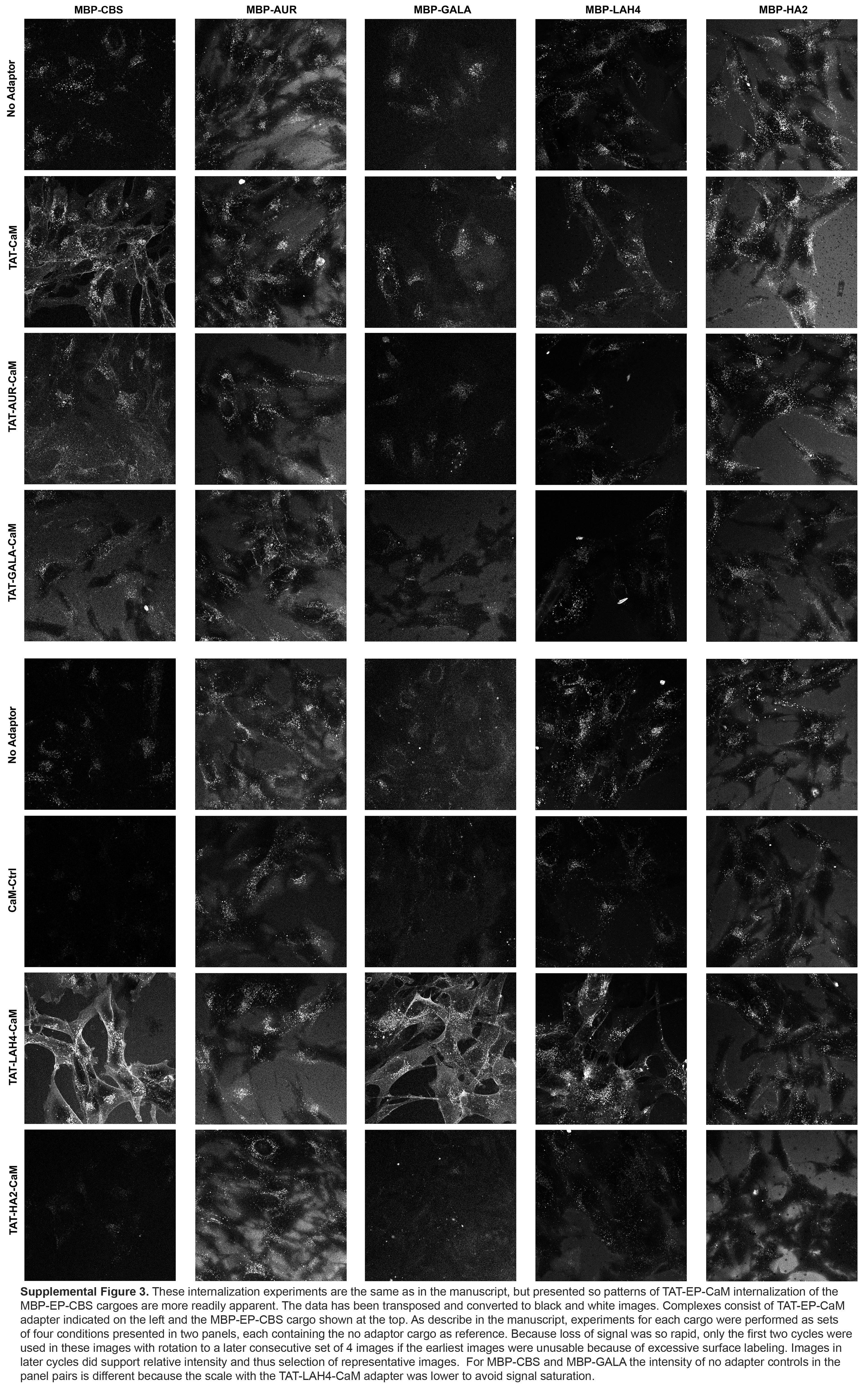

Supplement: Supplementary file 2 [file Image2.TIF]

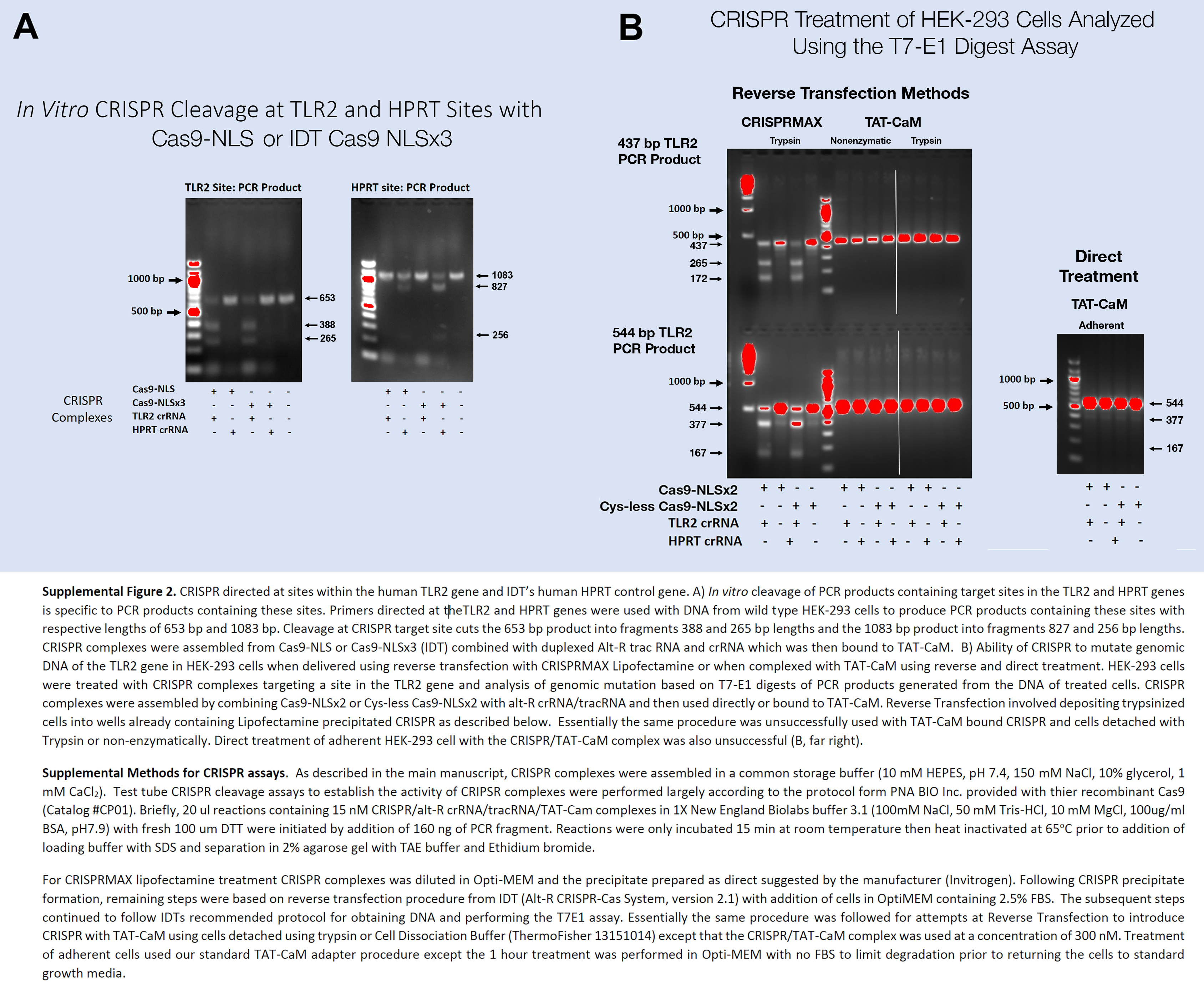

Supplement: Supplementary file 3 [file Image1.TIF]
