# Supplementary material for: A reversible cell penetrating peptide-cargo linkage allows dissection of cell penetrating peptide- and cargo-dependent effects on internalization and identifies new functionalities of putative endolytic peptides
Source: Front Pharmacol. 2022 Nov 21;13:1070464. doi: 10.3389/fphar.2022.1070464 (PMC9720253; doi:10.3389/fphar.2022.1070464)
Supplement: Supplementary file 4 [file DataSheet1.PDF]

TAT-CaM Constructs

All plasmids encoding TAT-CaM constructs are pET19b (EMD Millipore) derivatives with a synthetic gene encoding the TAT-CaM sequence cloned into the BamHI and HindIII sites. The resultant expressed protein thus has a vector-derived initiating Met followed by sequence encoding a His-tag, intervening SSGHI sequence prior to an enterokinase cleavage site and spacer sequence N-terminal to the encoded TAT-CaM or TAT-EP-CaM fusion protein. Tags were not removed prior to assays. Block sizes in the below schematics do not indicate relative residue numbers.

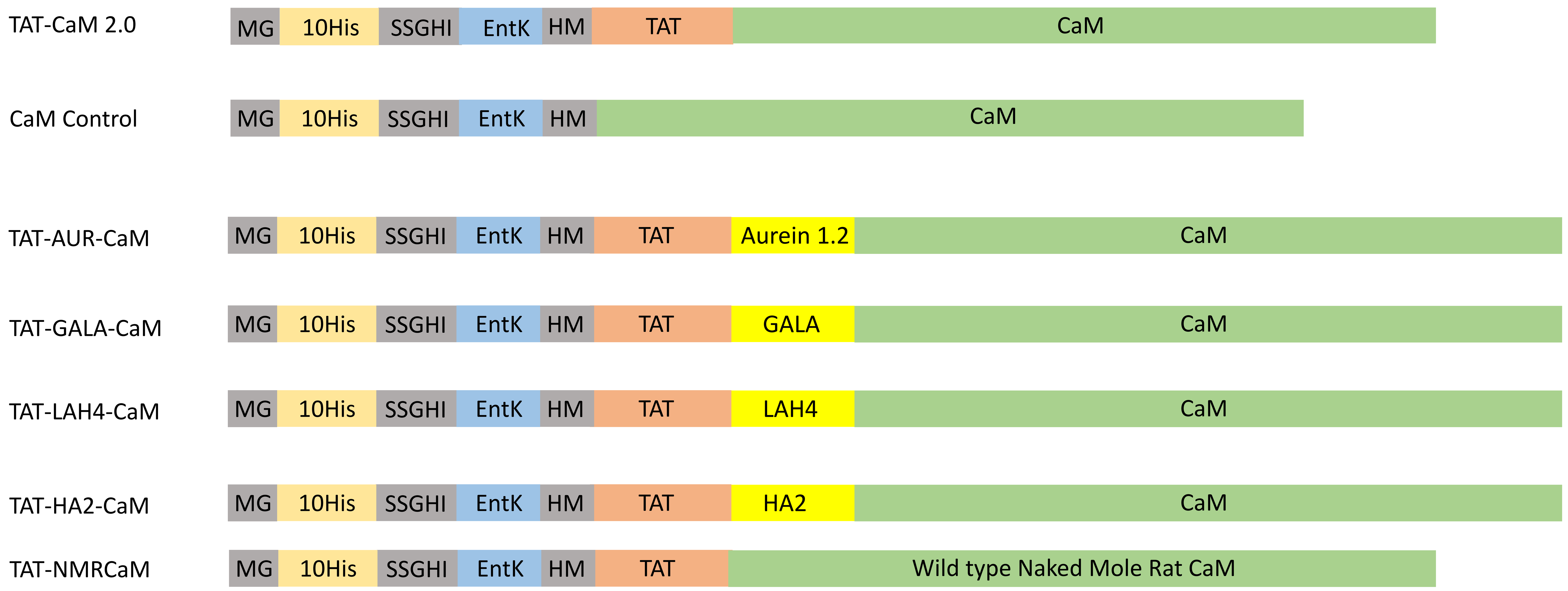

Amino acid sequence of calmodulin (Calmodulin-2, Isoform-2, human):

MADQLTEEQIAEFKEAFSLFDKDGDGTITTKE LGTVMRSLGQNPTEAELQDMINEVDADGNGTIDFPEFLTMMARKMKDTDSEEEI
REAFRVFDKDGNGYISAAELRHVMTNLGEKLTDEEVDEMIREADIDGDGQVNYEEFVQMMTAK

KEY

YGRKKRRQRRR: minimal TAT CPP sequence

L - Linker amino acids

DDDDK: Enterokinase cleavage site EntK

GALA: WEAALAEALAEALAEHLAEALAEALEALAA

Aurien 1.2: GLFDIIKKIAESF

HA2: GLFGAIAGFIEGGWTGMIDGWYG

LAH4: KKALLALALHHLAHLALHLALALKKA

pGFP (GFP+36 CaM Construct)

The plasmid encoding pGFP-CaM was similarly derived from pET19b, but into the NcoI and BamHI sites, which eliminate the HIS-tag and other vector-encoded sequences, leaving the initiating MG followed by the 6xHis-tagged GFP sequence from Thompson et al (2012) and then calmodulin. Block sizes in the schematic do not indicate relative residue numbers.

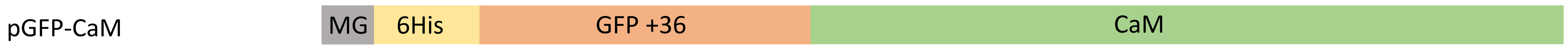

Amino acid sequence encoded by the synthetic gene with His-tag shown in black, +36GFP in orange, and CaM in green:

**HHHHHHGGASKGERLFRGKVPILVELKGDVNGHKFSVRGKGKGDATRGKLTLKFICTTGKLPVPWPTLVTTLTYGVQCFSRYPKHMK
RHDFFKSAMPKGYVQERTISFKKDGKYKTRAEVKFEGRTLVNRIKLKGRDFKEKGNILGHKLRYNFNSHKVYITADKRKNGIKAKFK
IRHNVKDGSVQLADHYQQNTPIGRGPVLLPRNHYLSTRSKLSKDPKEKRDHMVLLEFVTAAGIKHGRDERYKMADQLTEEQIAEFKE
AFSLFDKDGDGTITTKELGTVMRSLGQNPTEAELQDMINEVDADGNGTIDFPEFLTMMARKMKDTDSEEEIREAFRVFDKDGNGYIS
AAELRHVMTNLGEKLTDEEVDEMIREADIDGDGQVNYEEFVQMMTAK**

Cysless Tamavidin Construct

The plasmid encoding CBS-Tamavidin was derived from pCal-n-FLAG (Agilent Technologies) and contains a synthetic gene encoding tamavidin (C627S) cloned into the BamHI and HindIII sites of the vector. The resultant protein has vector-derived sequence containing an initiating MET, a calmodulin binding site from myosin light chain kinase, thrombin cleavage site and a FLAG tag N-terminal to Tam. A C-terminal 6xHis tag is also present prior to the stop codon. Block sizes in the schematic do not indicate relative residue numbers.

Tam-CBS (C627S)

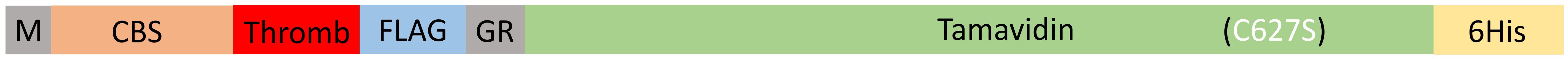

Tamavidin Sequence:

MSDVQSSLTGTWYNELNSKMELTANKDGTLTGKYLSKVGDVYVPYPLSGRYNLQPPAGQGVALGWAVSWE
NSKIHSATTWSGQFFSESSPVILTQWLLSSSTARGDVWESTLVGNDSFTKTAPTEQQIAHAQLHCRAPRLK

KEY

KRRWKKNFIAVSAANRFKKISSSGAL: CBS sequence is from myosin light chain kinase.

HHH: Histidines

LVPRGS: Thrombin Cleavage Site

DYKDDDDK: Flag Tag

DDDDK: Enterokinase cleavage site EntK

L - Linker or added amino acids

(WHITE): Deleted Or Altered amino acids

Maltose Binding Protein in pMAL-c5X

Plasmids encoding CBS-tagged maltose binding protein (MBP) cargos were derived from pMal-c5x (New England Biolabs). MBP sequence is endoded 5' to a vector-derived spacer sequence (noted as MBP+, below) upstream of the MCS into which a synthetic gene encoding the CBS or EP + CBS sequences shown was cloned via the BamHI and HindIII sites. A 6xHis tag and stop codon were included in the synthetic gene inside the HindIII site to eliminate vector-derived C-terminal sequence from the resultant protein. Block sizes in the below schematics do not indicate relative residue numbers.

MBP-CBS MBP^+ - KRRWKKNFIAVSAANRFKKISSSGAL PGAAHY HHHHHH *

MBP-AUR MBP^+ - GLFDIIKKIAESF KRRWKKNFIAVSAANRFKKISSSGAL PGAAHY HHHHHH *

MBP-GALA MBP^+ - WEAALAEALAEALAEHLAEALAEALEALAA KRRWKKNFIAVSAANRFKKISSSGAL PGAAHY HHHHHH*

MBP-LAH4 MBP^+ - KKALLALALHHLAHLALHLALALKKA KRRWKKNFIAVSAANRFKKISSSGAL PGAAHY HHHHHH *

MBP-HA2 MBP^+ - GLFGAIAGFIEGGWTGMIDGWYG KRRWKKNFIAVSAANRFKKISSSGAL PGAAHY HHHHHH *

KEY

MBP^+ = MBP plus NSSSNNNNNNNNNNLG IEGR ISHM…Insert… then at end of insert a stop *

KRRWKKNFIAVSAANRFKKISSSGAL: CBS sequence is from myosin light chain kinase.

HHH: Histidine

IEGR: Factor Xa

L - Linker Sequences

AUR (Aurien 1.2): GLFDIIKKIAESF

GALA: WEAALAEALAEALAEHLAEALAEALEALAA

LAH4: KKALLALALHHLAHLALHLALALKKA

HA2: GLFGAIAGFIEGGWTGMIDGWYG

Cas9 Constructs

Plasmids encoding CBS-Cas9 constructs are pET19b (EMD Millipore) derivatives with a synthetic gene encoding the CBS-Cas9 sequence cloned into the BamHI and HindIII sites. The resultant expressed proteins thus have a vector-derived initiating Met followed by sequence encoding a His-tag, intervening SSGHI sequence prior to an enterokinase cleavage site and spacer sequence N-terminal to the encoded CBS-Cas9 with or without NLS(s). Tags were not removed prior to assays. Block sizes in the below schematics do not indicate relative residue numbers.

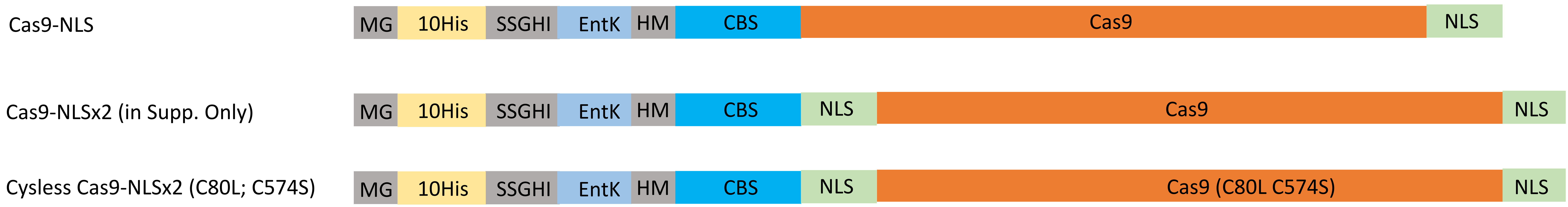

The sequence of the synthetic gene is shown below with CBS in red and Cas9 in black. The NLS sequences, when present, are shown in purple.

KRRWKKNFIAVSAANRFKKISSSGALPKKKRKVMDKKYSIGLDIGTNSVGWAVITDEYKVPSKKFKVLGNTDRHSIKKNLIGALLFDSGETAEATRLKRTARRRYTRRKNRICYLQEIFSNEMAKVDDS
FFHRLEESFLVEEDKKHERHPIFGNIVDEVAYHEKYPTIYHLRKKLVDSTDKADLRLIYLALAHMIKFRGHFLIEGDLNPDNSDVDKLFIQLVQTYNQLFEENPINASGVDAKAILSARLSKSRRLENLIA
QLPGEKKNGLFGNLIALSLGLTPNFKSNFDLAEDAKLQLSKDTYDDDLDNLLAQIGDQYADLFLAAKNLSDAILLSDILRVNTEITKAPLSASMIKRYDEHHQDLTLLKALVRQQLPEKYKEIFFDQSKN
GYAGYIDGGASQEEFYKFIKPILEKMDGTEELLVKLNREDLLRKQRTFDNGSIPHQIHLGELHAILRRQEDFYPFLKDNREKIEKILTFRIPYYVGPLARGNSRFAWMTRKSEETITPWNFEEVVDKGAS
AQSFIERMTNFDKNLPNEKVLPKHSLLYEYFTVYNELTKVKYVTEGMRKPAFLSGEQKKAIVDLLFKTNRKVTVKQLKEDYFKKIECFDSVEISGVEDRFNASLGTYHDLLKIIKDKDFLDNEENEDILE
DIVLTLTLFEDREMIEERLKTYAHLFDDKVMKQLKRRRYTGWGRLSRKLINGIRDKQSGKTILDFLKSDGFANRNFMQLIHDDSLTFKEDIQKAQVSGQGDSLHEHIANLAGSPAIKKGILQTVKVVD
ELVKVMGRHKPENIVIEMARENQTTQKGQKNSRERMKRIEEGIKELGSQILKEHPVENTQLQNEKLYLYYLQNGRDMYVDQELDINRLSDYDVDHIVPQSFLKDDSIDNKVLTRSDKNRGKSDNV
PSEEVVKKMKNYWRQLLNAKLITQRKFDNLTKAERGGLSELDKAGFIKRQLVETRQITKHVAQILDSRMNTKYDENDKLIREVKVITLKSKLVSDFRKDFQFYKVREINNYHHAHDAYLNAVVGTALI
KKYPKLESEFVYGDYKVYDVRKMIAKSEQEIGKATAKYFFYSNIMNFFKTEITLANGEIRKRPLIETNGETGEIVWDKGRDFATVRKVLSMPQVNIVKKTEVQTGGFSKESILPKRNSDKLIARKKDWD
PKKYGGFDSPTVAYSVLVVAKVEKGKSKKLKSVKELLGITIMERSSFEKNPIDFLEAKGYKEVKKDLIIKLPKYSLFELENGRKRMLASAGELQKGNELALPSKYVNFLYLASHYEKLKGSPEDNEQKQLF
VEQHKHYLDEIIEQISEFSKRVILADANLDKVLSAYNKHRDKPIREQAENIIHLFTLTNLGAPAAFKYFDTTIDRKRYTSTKEVLDATLIHQSITGLYETRIDLSQLGGDPKKKRKV
